# Nitrate Reductase NarGHJI Modulates Virulence via Regulation of *agr* Expression in Methicillin-Resistant *Staphylococcus aureus* Strain USA300 LAC

**DOI:** 10.1128/spectrum.03596-22

**Published:** 2023-05-18

**Authors:** Yujie Li, Ting Pan, Ruobing Cao, Wei Li, Zhien He, Baolin Sun

**Affiliations:** a Department of Oncology, The First Affiliated Hospital, University of Science and Technology of China, Hefei, People’s Republic of China; b Department of Life Science and Medicine, University of Science and Technology of China, Hefei, People’s Republic of China; University of Maryland School of Pharmacy

**Keywords:** nitrate reductase, NarGHJI, *Staphylococcus aureus*, *agr* system, cytokine, alpha-hemolysin, hemolysis, phenol-soluble modulin, virulence

## Abstract

Staphylococcus aureus is a pathogenic bacterium with a widespread distribution that can cause diverse severe diseases. The membrane-bound nitrate reductase NarGHJI serves respiratory function. However, little is known about its contribution to virulence. In this study, we demonstrated that *narGHJI* disruption results in the downregulation of virulence genes (e.g., *RNAIII*, *agrBDCA*, *hla*, *psmα*, and *psmβ*) and reduces the hemolytic activity of the methicillin-resistant S. aureus (MRSA) strain USA300 LAC. Moreover, we provided evidence that NarGHJI participates in regulating host inflammatory response. A mouse model of subcutaneous abscess and Galleria mellonella survival assay demonstrated that the *ΔnarG* mutant was significantly less virulent than the wild type. Interestingly, NarGHJI contributes to virulence in an *agr*-dependent manner, and the role of NarGHJI differs between different S. aureus strains. Our study highlights the novel role of NarGHJI in regulating virulence, thereby providing a new theoretical reference for the prevention and control of S. aureus infection.

**IMPORTANCE** Staphylococcus aureus is a notorious pathogen that poses a great threat to human health. The emergence of drug-resistant strains has significantly increased the difficulty of preventing and treating S. aureus infection and enhanced the pathogenic ability of the bacterium. This indicates the importance of identifying novel pathogenic factors and revealing the regulatory mechanisms through which they regulate virulence. The nitrate reductase NarGHJI is mainly involved in bacterial respiration and denitrification, which can enhance bacterial survival. We demonstrated that *narGHJI* disruption results in the downregulation of the *agr* system and *agr*-dependent virulence genes, suggesting that NarGHJI participates in the regulation of S. aureus virulence in an *agr*-dependent manner. Moreover, the regulatory approach is strain specific. This study provides a new theoretical reference for the prevention and control of S. aureus infection and reveals new targets for the development of therapeutic drugs.

## INTRODUCTION

Staphylococcus aureus is a Gram-positive, globally prevalent opportunistic pathogen that can cause a wide range of infections in various hosts (1, 2). To ensure its survival, S. aureus has evolved a comprehensive regulatory network to govern virulence, which includes quorum-sensing systems (QSSs), two-component systems (TCSs), alternative sigma factors (e.g., SigB), the SarA protein family, and regulatory RNA (3, 4). The accessory gene regulator (*agr*), a QSS, is one of the most well-researched regulatory systems; it consists of two adjacent transcripts (RNAII and RNAIII) driven by the P2 and P3 promoters, respectively (5). RNAII encodes the *agrBDCA* operon, whereas RNAIII encodes the major effector that regulates the expression of *agr*-dependent target genes *via* an antisense mechanism (3, 6). When the concentration of autoinducing peptide (AIP; encoded by *agrD* and transported into the extracellular space by AgrB) reaches a critical level, the histidine kinase AgrC undergoes autophosphorylation, and the phosphate is then relayed to AgrA, which can directly regulate the expression of RNAII and RNAIII by binding the P2 and P3 promoters (3, 7). The *agr* system is required for S. aureus infection and can regulate virulence factor expression in both RNAIII-dependent and -independent manner (7).

S. aureus produces various pathogenic factors that interfere with host immunity, including toxins, enzymes, cell surface-associated antigens, and immune evasion factors (3, 8). Toxins constitute a type of numerous virulence factors produced by S. aureus and are important components of its pathogenesis (8–13). The main toxins of S. aureus can be divided into three categories: pore-forming toxins (PFTs), exfoliative toxins, and superantigens (SAgs) (8). PFTs, which ultimately cause host cell lysis, can be subdivided into four types: alpha-hemolysins (Hla or alpha-toxin), beta-hemolysins, leukotoxins, and phenol-soluble modulins (PSMs) (8, 14). PSMαs and PSMβs are produced by the *psmα* and *psmβ* operons, respectively, and the delta-toxin (Hld) is encoded by *RNAIII* (15, 16). The expression of Hla and PSMs is tightly regulated by global virulence regulators. sRNA RNAIII, SarA, and Sae positively regulate *hla* expression, whereas Rot and SarT negatively regulate its expression (7, 17–21). In addition, AgrA positively regulates *psm* expression by directly binding to its promoter (7, 22).

The uptake of nitrogen is crucial for the survival of some bacteria (23). Reduction of nitrate to nitrite is mediated by membrane-bound nitrate reductase, which plays a crucial role in the nitrogen cycle and is encoded by the NarGHJI operon found in several bacteria (24). This process can occur under both anaerobic and aerobic conditions (25, 26). Weber et al. showed that disruption of nitrate reductase in Mycobacterium bovis significantly reduced the virulence but did not elucidate the mechanism (26). To the best of our knowledge, there are few reports so far on NarGHJI regulating the expression of virulence genes in bacterial pathogens.

In this study, we performed the targeted deletion of *narG* to investigate the contribution of NarGHJI to S. aureus virulence. Our findings revealed a novel role of NarGHJI in modulating S. aureus virulence via regulation of *agr* expression. Moreover, we provided evidence that NarGHJI participates in virulence regulation between different strains as a distinct “working model.” This study aimed to explore the mechanism through which NarGHJI contributes to the virulence of S. aureus, thereby providing a new theoretical reference for the treatment of S. aureus infection and highlighting a possible target for the development of new therapeutic drugs.

## RESULTS

### NarGHJI-mediated hemolytic activity of the MRSA strain USA300 LAC.

**FIG 1:**
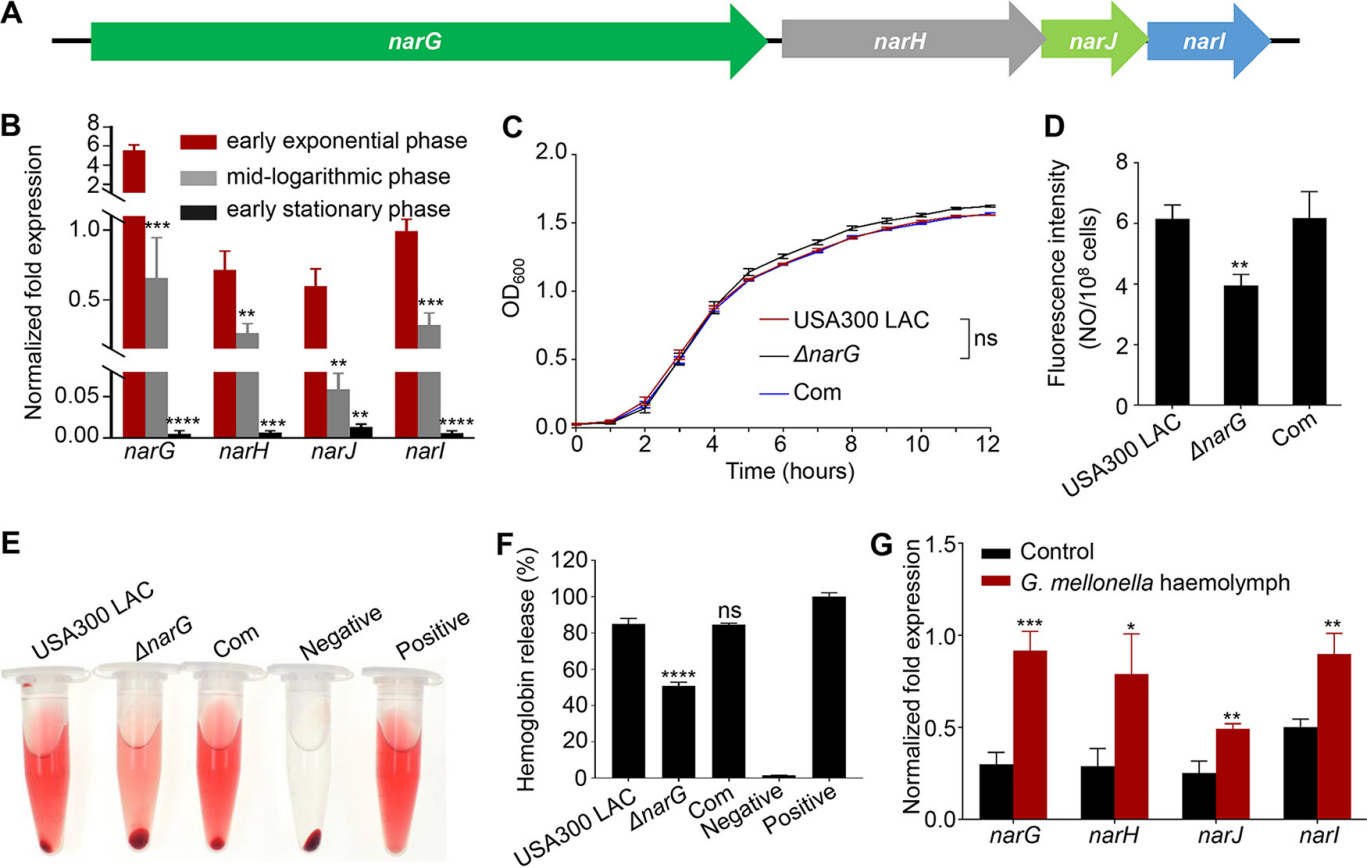
*narGHJI* disruption results in significantly reduced hemolytic activity. (A) Model of the nitrate reductase operon in S. aureus. (B) Transcription assays of *narG*, *narH*, *narJ*, and *narI* in different growth phases of S. aureus USA300 LAC (wild type). All experiments were performed in triplicate. The data are represented as means ± the standard deviations (SD). **, *P < *0.01; ***, *P < *0.001; ****, *P < *0.0001 (two-tailed Student *t* test). (C) Comparison of the growth rates of wild type, *ΔnarG*, and *narG* chromosomal-complemented (Com) strains in TSB, with an initial OD_600_ of 0.05. All experiments were performed in triplicate. The data are represented as means ± the SD. ns, not significant. A two-tailed Mann-Whitney U test was used to analyze the difference of growth rate between the *ΔnarG* and USA300 LAC strains. (D) Detection of NO production in the wild type, *ΔnarG*, and complemented strains. The assays were performed in triplicate. The data are represented as means ± the SD. **, *P < *0.01 (two-tailed Student *t* test). (E and F) Hemolytic activities of the wild type, *ΔnarG*, and chromosomal-complemented strains. Hemolytic activities were determined by incubating the target samples with 3% rabbit erythrocytes, phosphate-buffered saline (PBS, negative control), or ddH_2_O (positive control, 100% hemolytic activity) for 30 min (E), and the percentage of released hemoglobin (relative to the positive control) was determined by measuring the absorbance of supernatants at 543 nm (F). All experiments were performed in triplicate. The data are represented as means ± the SD. ****, *P < *0.0001 (two-tailed Student *t* test). ns, not significant. (G) The expression of *narGHJI* was induced by G. mellonella hemolymph. The target strains (early exponential phase) were incubated with G. mellonella hemolymph at 37°C for 30 min. All experiments were performed in triplicate. The data are represented as means ± the SD. *, *P < *0.05; **, *P < *0.01; ***, *P < *0.001 (two-tailed Student *t* test).

The S. aureus nitrate reductase operon contains four genes in the order *narG narH narJ narI* (*narGHJI*, clustered together in an operon; Fig. 1A). Reverse transcription-quantitative PCR (RT-qPCR) data indicated that the expression of all four genes was higher in the early exponential phase than in the midlogarithmic and early stationary phases (Fig. 1B). A *narGHJI* mutant was constructed via the targeted deletion of *narG* in MRSA USA300 LAC to investigate the function of NarGHJI in S. aureus. The growth curves indicated that *narGHJI* disruption had no significant effect on S. aureus growth (Fig. 1C). A previous study suggested that NarGHJI participates in nitrate assimilation of M. tuberculosis (23). Our results showed that the disruption of *narGHJI* dramatically reduced NO production (Fig. 1D), consistent with the findings in M. tuberculosis. The hemolytic activities of wild type, *ΔnarG*, and complementation strains were quantitatively assessed using rabbit erythrocytes to determine whether NarGHJI is involved in regulating the hemolytic activity of S. aureus. The results indicated that the hemolytic activity of the *ΔnarG* mutant significantly decreased by 83.5% compared to that of the wild type, and the defect could be restored by the chromosomal-complemented strain (Fig. 1E and F). Intriguingly, we also discovered that the hemolymph of Galleria mellonella, which is considered a potential infection model, stimulated the expression of *narG*, *narH*, *narJ*, and *narI* (Fig. 1G), suggesting a crucial role of NarGHJI in the regulation of S. aureus virulence.

### NarGHJI positively regulates the transcription of virulence genes.

**FIG 2:**
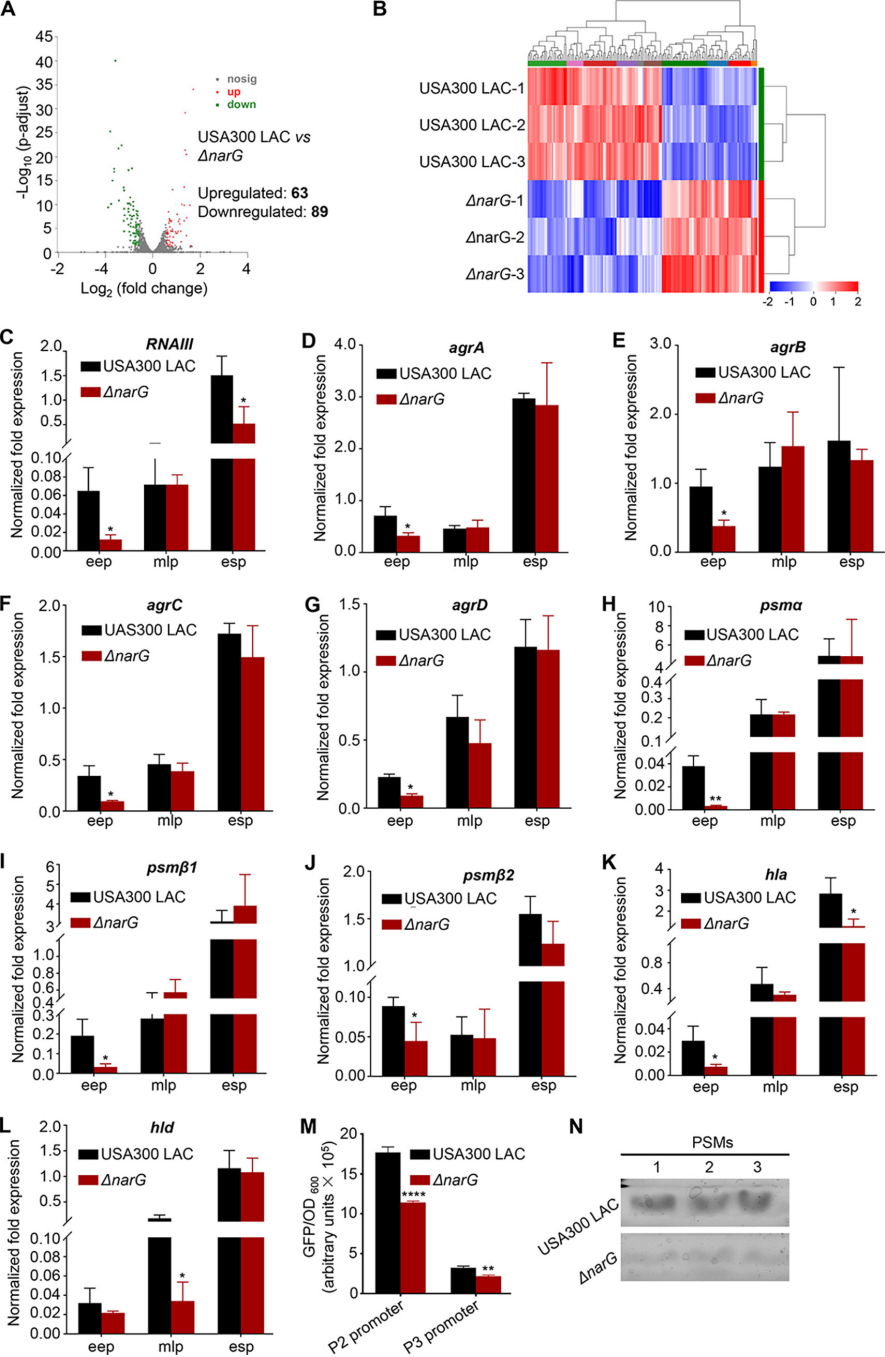
*narGHJI* disruption decreases the expression of virulence genes. (A) DEGs between USA300 LAC and *ΔnarG* strains. (B) Pairwise Euclidean distance (heat map) of the transcriptional profiles of the tested samples. (C to L) Detection of the expression of virulence genes via using RT-qPCR. All experiments were performed in triplicate. The data are represented as means ± the SD. *, *P < *0.05; **, *P < *0.01 (two-tailed Student *t* test for panels D, E, F, H, I, K, and L and two-tailed Mann-Whitney U test for panels C, G, and J). eep, early exponential phase; mlp, midlogarithmic phase; esp, early stationary phase. (M) Activity determination of the P2 and P3 promoters. The data are represented as means ± the SD. **, *P < *0.01; ****, *P < *0.0001 (two-tailed Student *t* test). (N) Production of PSMs in *ΔnarG* and USA300 LAC strains. The production of PSM peptides was measured by using Coomassie blue-stained 12% SDS-PAGE.

S. aureus has evolved a complex regulatory network of virulence expression (3). RNA sequencing (RNA-seq) was performed to determine whether NarGHJI alters the expression of virulence factors. The results indicated that the gene expression profiles of S. aureus USA300 LAC and *ΔnarG* strains were considerably different, with 63 upregulated and 89 downregulated genes (Fig. 2A). Differentially expressed genes (DEGs) among three biological replicates were displayed using Euclidean distance calculation (Fig. 2B). Among the significantly altered genes, the downregulated genes *agrA* and *agrB* were noted in USA300 LAC relative to those in the *ΔnarG* mutant (see Fig. S1A in the supplemental material). To further illustrate whether *narGHJI* is involved in regulating the expression of the *agr* system, we performed RT-qPCR to detect the transcription of *RNAII*, an operon of four genes (*agrBDCA*), and *RNAIII*, the major effector of the *agr* system. As expected, we found that the disruption of *narGHJI* significantly decreased the expression of *agrBDCA* and *RNAIII* (Fig. 2C to G). Furthermore, the absence of significant alterations in the transcription of other regulatory systems, such as *saeR*, *sarA*, and *sigB*, in different growth stages suggests that *narGHJI* may specifically regulate the *agr* system (see Fig. S1B). For further confirmation, we detected the transcription of virulence factors that are strictly regulated by the *agr* system. The results revealed that the expression of *psmα*, *psmβ1*, *psmβ2*, *hla*, and *hld*, but not *hlb* and *hlgC*, was considerably lower in the *ΔnarG* mutant than in S. aureus USA300 LAC (Fig. 2H to L; see also Fig. S1C and D in the supplemental material). The expression of virulence factors significantly decreased in the early exponential phase, which was consistent with the finding that *narGHJI* expression peaked in the early exponential phase (Fig. 1B). In addition, we also found that overexpression of *narGHJI* significantly increased the expression of *RNAIII*, *agrA*, and *agrC* (see Fig. S2A and B). Considering that the above S. aureus samples were obtained from tryptic soy broth (TSB) culture, to exclude the interference from rich medium, S. aureus samples from the basic medium RPMI 1640 were collected for the transcriptional expression analysis of virulence genes. The results indicated that the expression of almost all the genes we examined was significantly downregulated in the *ΔnarG* mutant in the early exponential and midlogarithmic phases compared to the wild type (see Fig. S3A to J). Next, we determined the activities of the P2 and P3 promoters using a green fluorescent protein (GFP) reporter system, and the results indicated that these activities were remarkably weaker in the *ΔnarG* mutant than in the wild type (Fig. 2M). We also found that the production of PSM peptides was obviously lower in the *ΔnarG* mutant than in the wild type (Fig. 2N). Taken together, the results suggest that *narGHJI* regulates virulence genes through specific regulation of *agr* system expression.

### NarGHJI participates in the regulation of AIP-I production.

**FIG 3:**
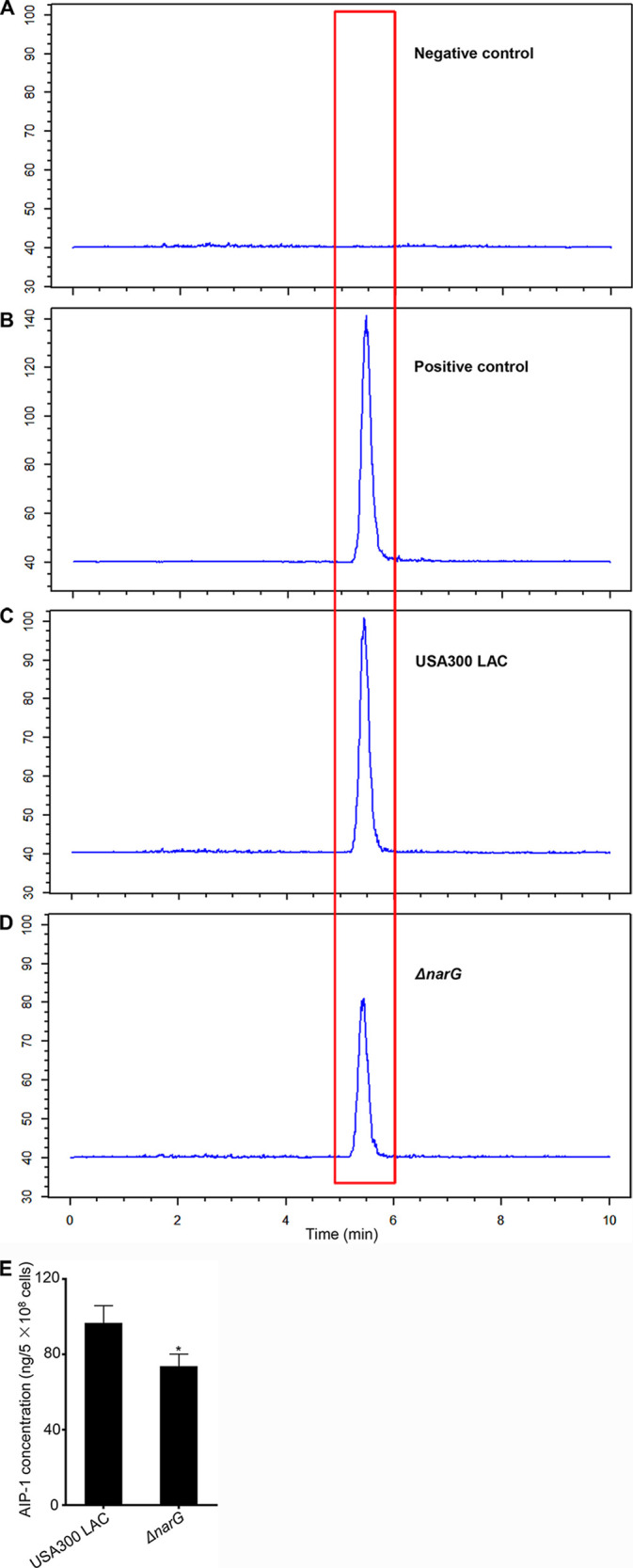
AIP-I production by the wild type and *ΔnarG* strains. (A to D) Chromatograms of the negative control (A, TSB), the positive control (B, standard AIP-I, 1000 ng/mL), USA300 LAC (C), and the *ΔnarG* mutant (D). (E) AIP-I quantitation in USA300 LAC and the Δ*narG* mutant. All experiments were performed in triplicate. The data are represented as means ± the SD. *, *P < *0.05 (two-tailed Student *t* test).

The AIP-dependent QSS contains a typical self-activation circuit encoded by the *agrBDCA* locus in S. aureus. When the concentration of AIP reaches a threshold, the sensor system is activated, resulting in the production of virulence factors, such as toxins, SAgs, and exoenzymes (27, 28). AIPs are categorized into four groups, namely, AIP-I to AIP-IV, and USA300 strains can produce AIP-I (27, 29). To verify whether *narGHJI* is involved in regulating AIP-I production, we performed liquid chromatography-mass spectrometry approach to detect the AIP-I concentration of each target strain. The results indicate that the deletion of *narG* results in significantly decreased AIP-I production (Fig. 3), suggesting a critical role of NarGHJI in regulating the expression of virulence factors through the *agr* system.

### *narGHJI* disruption results in decreased ability to induce cytokine production.

**FIG 4:**
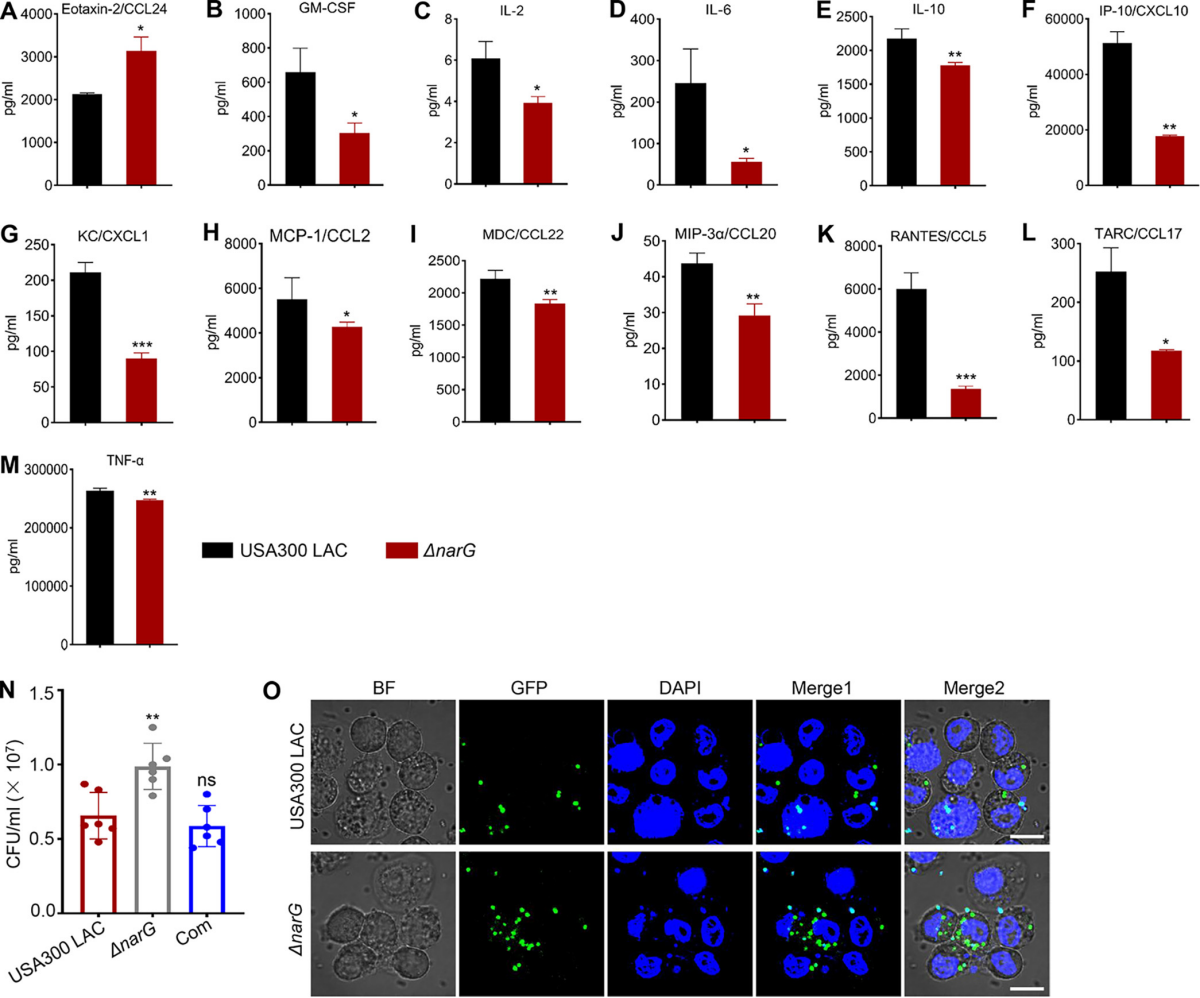
Cytokine/chemokine production and intracellular survival of S. aureus in host cells. (A to M) Determination of cytokine/chemokine levels using ELISA in the culture supernatant of S. aureus-treated mouse macrophage RAW 264.7 cells. RAW 264.7 cells were infected with S. aureus for 6 h at an MOI of 50. All experiments were performed in triplicate. The data are represented as means ± the SD. *, *P < *0.05; **, *P < *0.01; ***, *P < *0.001 (two-tailed Student *t* test for panels B to G, I to K, and M and two-tailed Mann-Whitney U test for panels A, H, and L). (N) Detection of the intracellular survival of S. aureus in RAW 264.7 cells (MOI = 50 for 6 h). The data are represented as means ± the SD from six independent experiments (*n* = 6). **, *P < *0.01 (two-tailed Student *t* test). ns, not significant. (O) Distribution of GFP-labeled S. aureus in RAW 264.7 cells. Nuclei were stained with DAPI. MOI = 50; coincubation time = 6 h. Scale bar, 5 μm.

It has been well established that inflammatory cytokines and chemokines play critical roles in inflammation during infection (30). The production of 31 cytokines/chemokines was examined in S. aureus-infected mouse macrophage RAW 264.7 cells to verify whether NarGHJI is involved in controlling the inflammatory response of host cells. Among them, the production of 12 cytokines/chemokines, such as granulocyte-macrophage colony-stimulating factor (GM-CSF), interleukin-6 (IL-6), IL-10, KC/CXCL1, tumor necrosis factor alpha (TNF-α), and IP-10/CXCL10, was significantly lower in *ΔnarG* mutant-infected cells than in USA300 LAC-infected cells (Fig. 4B to M). However, the production of 18 cytokines/chemokines, such as BCA-1/CXCL13, IL-1β, IL-4, gamma interferon (IFN-γ), SCYB16/CXCL16, and macrophage inflammatory protein 1α (MIP-α)/CCL3, was not significantly different between the groups (see Fig. S4A to R). Interestingly, the release of Eotaxin-2/CCL24, a member of the CC chemokine family, significantly increased in *ΔnarG* mutant-infected cells compared to that in USA300 LAC-infected cells (Fig. 4A). Notably, we also found that the deletion of *narG* significantly increased the intracellular survival of S. aureus in macrophages (Fig. 4N and O), indicating that NarGHJI may be a negative regulatory factor in immune evasion. Taken together, the results reveal that NarGHJI might be involved in regulating host inflammatory response.

### NarGHJI contributes to the virulence of *S. aureus* in mouse subcutaneous abscess and *G. mellonella* infection models.

**FIG 5:**
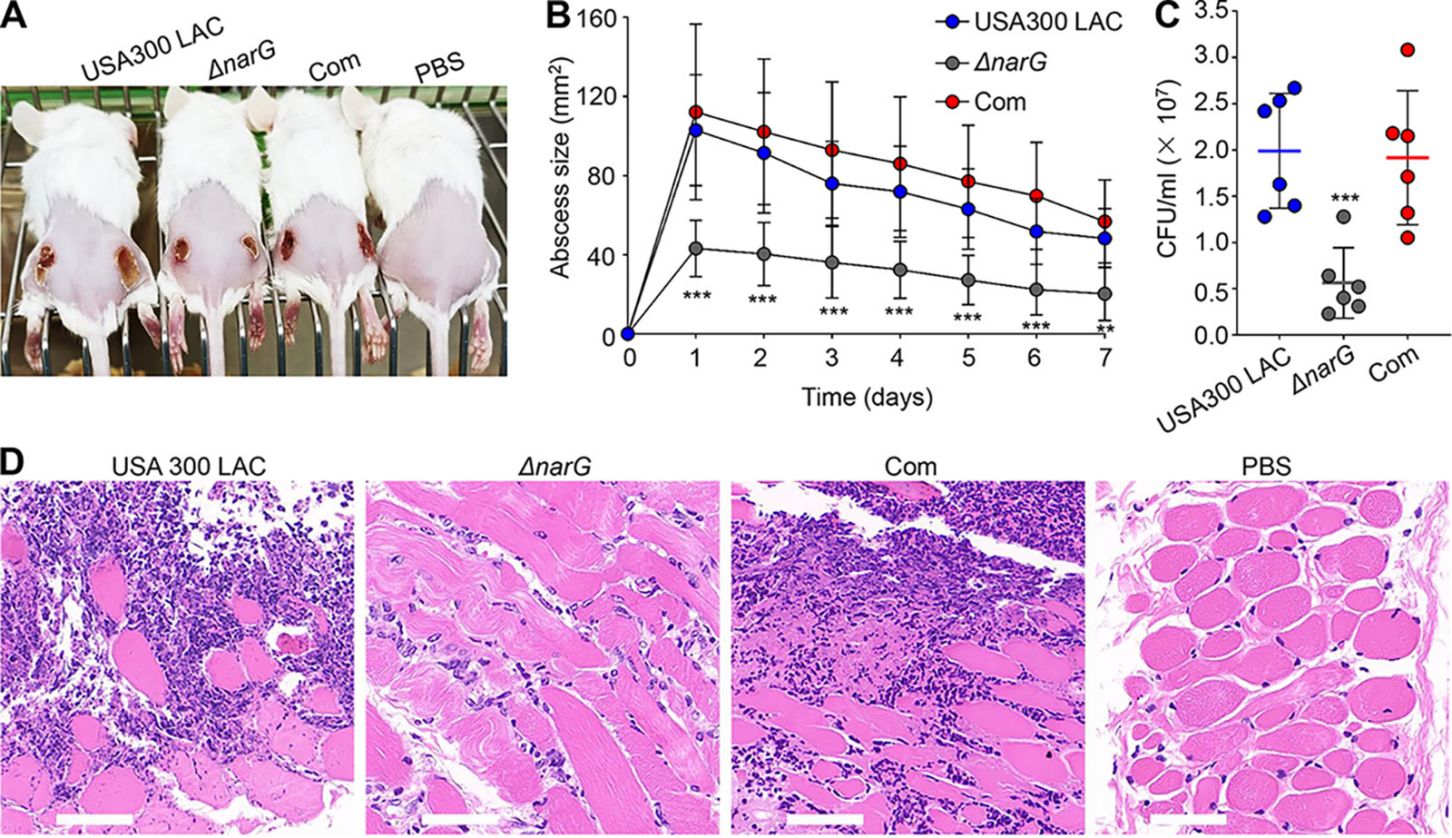
Deletion of *narG* reduces the virulence of S. aureus in a mouse subcutaneous abscess model. (A) Representative abscesses at day 7 after infection. The mice were inoculated with 50 μL of PBS containing 3 × 10^7^ CFU of each target strain, and PBS was used as a negative control. Abscess areas were assessed daily. *n* = 6 to 8. (B) Statistical representation of mouse subcutaneous abscess areas in panel A. **, *P < *0.01; ***, *P < *0.001 (two-tailed Mann-Whitney U test). (C) Determination of CFU in each abscess. ***, *P < *0.001 (two-tailed Student *t* test). (D) H&E staining of representative mouse abscesses. Scale bar, 50 μm.

According to previous studies, S. aureus skin and soft tissue infections are facilitated by the well-characterized toxins Hla and PSMs (15, 31). The finding that *narGHJI* regulates Hla and PSM expression through specific regulation of the *agr* system prompted us to determine whether *narGHJI* participates in the regulation of S. aureus virulence. To this end, mouse subcutaneous abscess and G. mellonella infection models were established to evaluate the virulence of S. aureus. The results indicated that the abscess areas caused by the *ΔnarG* mutant were significantly diminished compared to those caused by the USA300 LAC and chromosome-complemented strain (Fig. 5A and B). Similarly, bacterial colonization in the skin abscesses was considerably reduced in *ΔnarG*-infected mice compared to that in USA300 LAC-infected mice, and the defect was restored by chromosomal complementation (Fig. 5C). Skin abscesses caused by the *ΔnarG* mutant displayed less severe inflammation with leukocyte infiltration and destruction of the skin structure compared to those caused by USA300 LAC, based on histological examinations (hematoxylin and eosin [H&E] staining, Fig. 5D). We also found that *narGHJI* disruption resulted in decreased virulence using G. mellonella larvae as the target host (see Fig. S5). Altogether, these findings indicate that NarGHJI can positively contribute to the pathogenicity of S. aureus by controlling the expression of virulence genes.

### The contribution of NarGHJI to virulence of *S. aureus* is *agr* dependent and strain specific.

**FIG 6:**
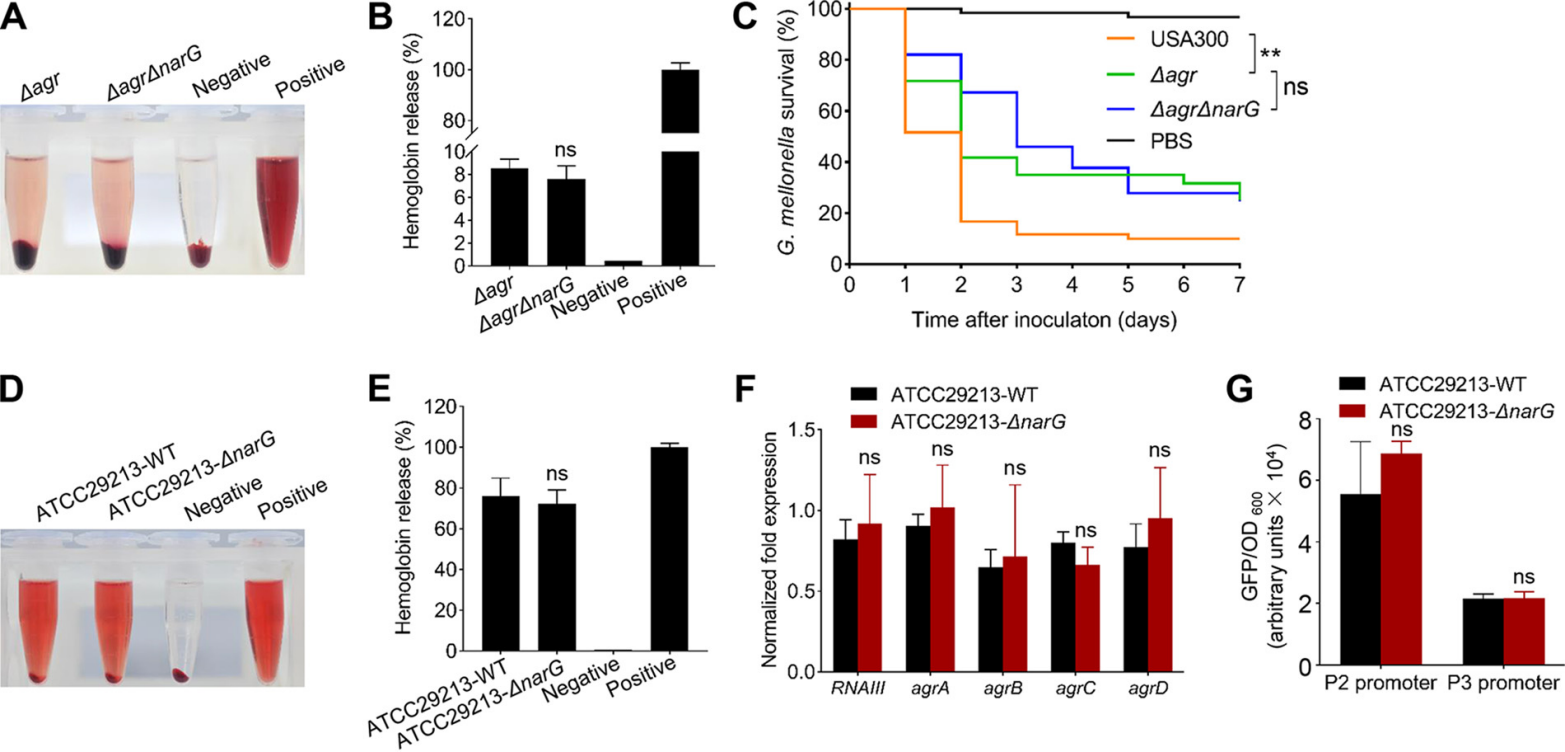
NarGHJI regulates the virulence of S. aureus in *agr*-dependent and strain-specific manners. (A, B, D, and E) Hemolytic activities of the target strains. For panels A and B, the hemolytic activities were determined by incubating the target samples with 15% rabbit erythrocytes, PBS (negative control), or ddH_2_O (positive control, 100% hemolytic activity) for 12 h (A), and the percentage of released hemoglobin (relative to the positive control) was determined by measuring the absorbance of supernatants at 543 nm (B). For panels D and E, the hemolytic activities were determined by incubating the target samples with 3% rabbit erythrocytes for 30 min. All experiments were performed at least in triplicate. The data are represented as means ± the SD and were evaluated using a two-tailed Student *t* test. ns, not significant. (C) Virulence detection of the target strains using G. mellonella infection model. G. mellonella larvae were inoculated with 1.5 × 10^6^ CFU of each target strain. Survival curves were constructed using GraphPad Prism 8, and the Mantel-Cox test was used to analyze the difference between two groups. **, *P < *0.01; ns, not significant. (F) Determination of the expression of virulence genes of the target strains. All samples were collected from the early exponential phase of the strains. The data are represented as means ± the SD and were evaluated using a two-tailed Student *t* test. ns, not significant. (G) Detection of the activities of the P2 and P3 promoters. The data are represented as means ± the SD and were evaluated using a two-tailed Mann-Whitney U test. ns, not significant.

To confirm whether NarGHJI regulates the virulence of S. aureus in an *agr*-dependent manner, an *agr* mutant was constructed in the *ΔnarG* mutant. The hemolytic activity of Δ*narG* and *agr* double mutant did not differ significantly from that of single Δ*agr* mutant (Fig. 6A and B). Corresponding to this, no statistically significant changes were observed in the virulence of the Δ*narG* mutant and the *narG* *agr* double mutant using G. mellonella larvae as the target host (Fig. 6C). Moreover, similar to the *ΔnarG* mutant, the production of some cytokines/chemokines, such as Eotaxin-2/CCL24, GM-CSF, IL-2, IL-6, IP-10/CXCL10, and TNF-α, significantly changed in *agr* mutant-infected cells compared to that in USA300 LAC-infected cells (see Fig. S7A to L). The Δ*agr* mutant caused almost no visible mouse subcutaneous abscess, leukocyte infiltration, and destruction of the skin structure (see Fig. S7M and N), indicating that the phenotypic changes caused by the *ΔnarG* mutant are intermediate relative to those between the *agr* mutant and USA300 LAC. We also found that the virulence of the *agr* mutant to G. mellonella could match that of the *ΔnarG* mutant (Fig. 6C). These results suggest that NarGHJI may play a critical role in the regulation of virulence in an *agr*-dependent manner. To investigate whether the contribution of NarGHJI to S. aureus virulence is strain specific, we knocked out *narG* in MRSA strain N315 and MSSA (methicillin-sensitive S. aureus) strain ATCC 29213. The results demonstrated that, compared to the wild type, the hemolytic activity of the *ΔnarG* mutant in N315 significantly decreased, whereas that in ATCC 29213 showed no visible change (Fig. 6D and E; see also Fig. S6A and B). Corresponding to these, we found that the disruption of *narGHJI* in N315, but not in ATCC 29213, significantly reduced the expression of *agrBDCA* and *RNAIII* and the activities of the P2 and P3 promoters (Fig. 6F and G; see also Fig. S6C and D). Altogether, these findings suggest that the virulence regulatory mechanism of NarGHJI is *agr* dependent and strain specific.

## DISCUSSION

**FIG 7:**
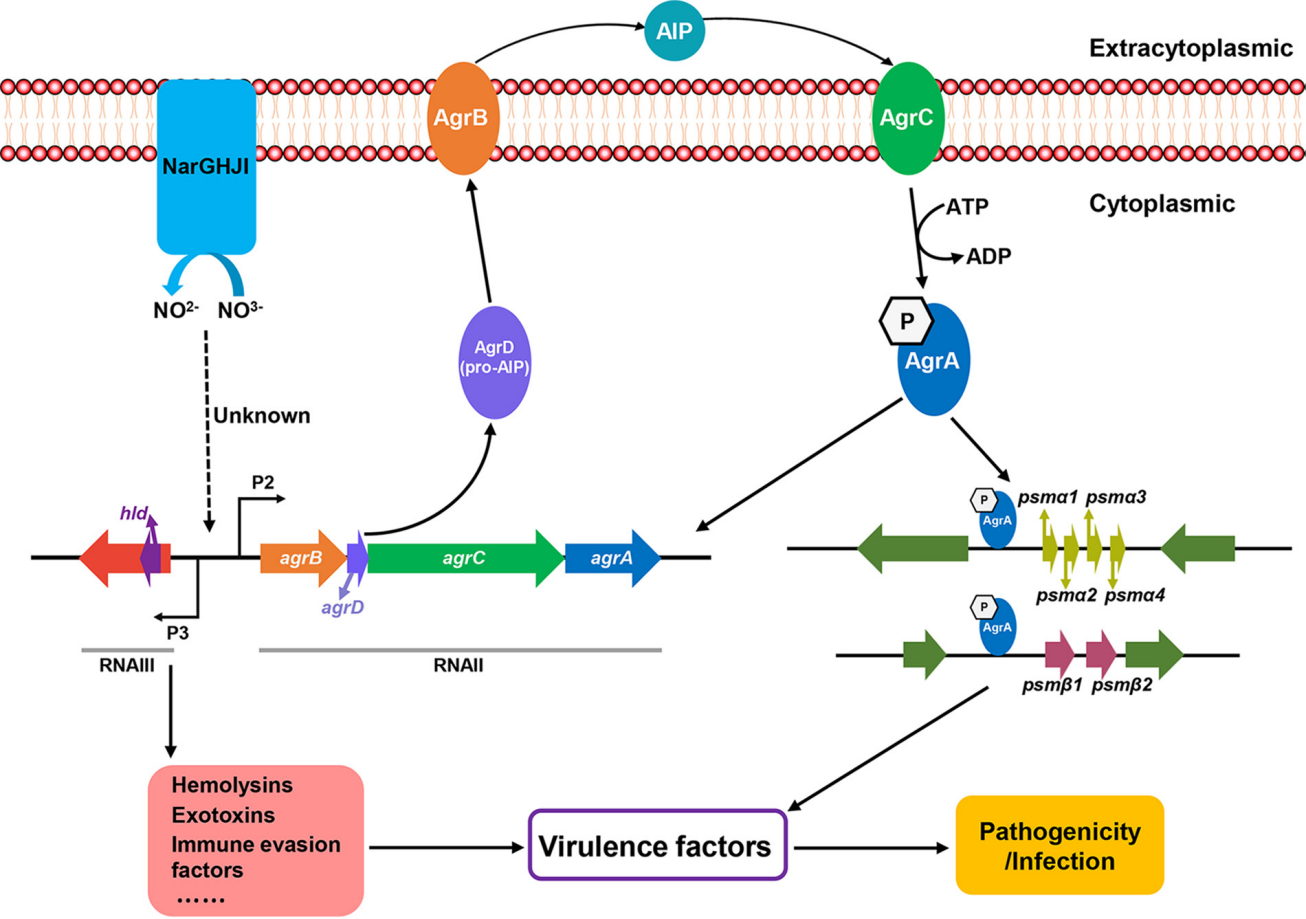
Hypothetical schematic model of the molecular organization and signal transduction of NarGHJI for regulating the pathogenicity of S. aureus.

S. aureus, a Gram-positive opportunistic pathogen, can cause various community- and hospital-acquired infections, including bacteremia, pneumonia, sepsis, and osteomyelitis (32, 33). The emergence of multidrug-resistant and highly virulent S. aureus has brought great challenges to the treatment of its infection. Elucidating the pathogenic mechanism of S. aureus can provide a theoretical reference for the treatment of S. aureus infection. Previous studies have shown that the nitrate reductase NarGHJI mediates the reduction of nitrate under both anaerobic and aerobic conditions, thereby enhancing bacterial survival (24–26). Nevertheless, little is known about the role of NarGHJI in regulating virulence. Here, we revealed that NarGHJI regulates virulence by governing *agr* expression. However, the mechanism underlying the regulation of NarGHJI to Agr remains to be investigated. The hypothetical pathway of the NarGHJI-mediated regulation of virulence is presented in Fig. 7.

The *agr* QSS, one of the well-studied virulence regulatory systems of S. aureus, plays a critical role in regulating the expression exotoxins and exoenzymes; moreover, it is required for virulence in animal models of skin infection, pneumonia, and endocarditis (3). *agr* expression is controlled by various factors, including SarA, SarU, AIP, ArlRS (a TCS), MgrA/Rat/NorR, and SvrA (a novel membrane protein) (3). The results we provided here that NarGHJI modulates virulence by regulating *agr* expression can enrich the knowledge of the virulence regulatory network of S. aureus.

In this study, we showed that the disruption of *narGHJI* significantly reduced the expression *agr* system and virulence genes that are regulated by it, and the changes occur mainly in the early exponential and midlogarithmic phases (Fig. 2C to L; see also Fig. S3A to J). This may be due to the higher expression of *narGHJI* in the early exponential and midlogarithmic phases, but lower expression in the early stationary phase (Fig. 1B). We also found that the differences in fold change of *agr*-related genes are modest. Agr is a global virulence gene regulator (34), so moderate alteration may cause significant change in virulence. Moreover, there may be an additive effect of these virulence genes in the regulation of S. aureus virulence. On the other hand, RNA-seq results indicated that *narGHJI* disruption results in upregulation of 63 genes and downregulation of 89 genes (see Table S3). Among them, in addition to *agr*, disruption of *narGHJI* also caused significant reductions in other genes, such as VraX, a secreted protein involved in the pathogenesis of S. aureus. *agr* may not be the only target of NarGHJI, and NarGHJI may also modulate S. aureus virulence by regulating *vraX* expression, but the mechanism underlying the regulation remains to be investigated.

S. aureus has become a serious threat to human health because of the emergence of drug-resistant strains and their adaptability to the host by evading the immune system (35). Approximately 40 immune evasion proteins, such as Hla, PSMs, and HlgC, have been identified and characterized (35, 36). We demonstrated that *narGHJI* disruption significantly reduced the expression of Hla and PSMs (Fig. 2H to K and N). However, the Δ*narG* mutant exhibited greater intracellular survival in RAW 264.7 cells (Fig. 4N and O). Combined with the result that no visible differences in growth curves were observed between the *ΔnarG* mutant and the wild type (Fig. 1C), the above-mentioned results revealed that the mutant has a greater ability to evade the immune system. Based on these findings, we hypothesized that the phenomenon of high intracellular survival of the *ΔnarG* mutant in host cells benefits from the expression of other immune evasion factors. We observed that the expression of gamma-hemolysin HlgC, another immune evasion factor, significantly increased in this strain (see Fig. S1D). Altogether, the phenotype of low-virulence and high-survival of the *ΔnarG* mutant implied that NarGHJI plays a critical role in S. aureus pathogenicity.

Many bacteria are able to utilize O_2_ and nitrate as alternative electron acceptors for respiration (37). Apart from oxygen, nitrate is the most widely used alternative electron acceptor, and therefore nitrate assimilation is essential for the survival of some bacteria (23, 38). S. aureus, a facultative anaerobe, is able to switch under anaerobic condition to nitrate respiration and fermentation (39, 40). It has been reported the ability of some bacteria to obtain nutrients in the infected tissue is limited, but it can assimilate nitrate (41, 42), which may be one of the reasons that NarGHJI promotes the virulence of S. aureus. Nitrate reductase NarGHJI is regulated by NreABC-system in Staphylococcus carnosus (43, 44). Schlag et al. showed that the deletion of *nreABC* did not affect the expression of virulence factors (45). In this study, however, we found that NarGHJI participates in regulating the expression of virulence genes, indicating that the pathway by which NarGHJI regulates the virulence of S. aureus may be novel and NreABC-system independent.

Knockout of *narG* in S. aureus MRSA strains USA300 LAC and N315 significantly reduced the pathogenicity, but not in MSSA strain ATCC 29213. Multilocus sequence typing analysis indicated that ATCC 29213 and N315 belong to ST5 and that USA300 LAC belongs to ST8 (see Table S4 [http://bacdb.cn/BacWGSTdb/analysis_multiple.php]). In addition, phylogenetic analysis showed that ATCC 29213 and N315 are closely related (see Fig. S8). These data suggest that the regulation of virulence by NarGHJI may be MRSA strain specific and that the contribution of NarGHJI to the virulence of different S. aureus strains has little to do with the differences between the genomes of these strains. In view of the sample size limitation, these findings need to be further explored. Anyway, our data denoted that the contribution of NarGHJI to S. aureus virulence is strain specific.

In conclusion, the nitrate reductase NarGHJI positively contributes to S. aureus virulence in an *agr*-dependent manner, and the virulence regulatory mechanism of NarGHJI is strain specific. These findings reveal a vital role of NarGHJI in regulating S. aureus pathogenicity, thereby providing a new theoretical reference for the treatment of S. aureus infection.

## MATERIALS AND METHODS

### Strains, plasmids, and culture conditions.

S. aureus wild type and mutant strains were cultured in TSB (211825; BD Difco) with shaking at 220 rpm or on tryptic soy agar at 37°C. Escherichia coli TOP10 (DL1010; Weidi) was used for routine DNA manipulation, and the derived strains were cultured in lysogeny broth (LB) with shaking at 220 rpm or on lysogeny broth agar (LA) at 37°C. According to the requirement, 60 μg/mL ampicillin sodium salt and 15 μg/mL chloramphenicol were added to TSB and LB, respectively. The strains and plasmids used in this study are listed in Table S1.

### Construction of Δ*narG* mutant, complementation, overexpression, and GFP reporter strains.

The primers used in this study are listed in Table S2. The *narG* knockout mutant was constructed using plasmid pBTs, as described previously (46). All PCR products were obtained using PrimeSTAR MAX Premix (R045; TaKaRa). The resultant products were cloned into target vectors using ClonExpress II (C112; Vazyme). Homologous recombination was performed to delete *narG*. To construct the vector, 5′-end 774-bp and 3′-end 783-bp fragments of *narG* were amplified via PCR using S. aureus genomic DNA as the template with the primer pairs *narG*-LB-F/*narG*-LB-R and *narG*-RB-F/*narG*-RB-R, respectively. The PCR products were integrated *via* overlap extension PCR and then inserted into pBTs using ClonExpress II (C112) to generate the vector pBTs-LB-*narG*-RB. The resulting vector was transformed into S. aureus strain RN4220 for modification and was subsequently transformed into S. aureus strain USA300 LAC. A similar approach was used to construct the complementation strain using the primers listed in Table S2. The resulting vector (pBTs-*narG*-Com) was transformed into RN4220 for modification and was subsequently transformed into the *ΔnarG* mutant. Mutant colonies were subjected to single-cell isolation, and the accurately integrated strains were verified via PCR and RT-qPCR. The construction of the Δ*narG* mutant in N315 and ATCC 29213, as well as *agr* mutant in the target strains, was similar to that of the Δ*narG* mutant in USA300 LAC.

For construction of *narGHJI* overexpression strain, the fragment for overexpression was amplified by using PCR with the primer pair *narGHJI*-OE-F/*narGHJI*-OE-R and then inserted into PLI50 using ClonExpress II (C112) to generate the vector PLI50-*narGHJI*-OE. The resulting vector was transformed into S. aureus strain RN4220 for modification and was subsequently transformed into S. aureus strain USA300 LAC.

To construct the GFP reporter vector, the P2 (281-bp) and P3 (228-bp) promoters were cloned from USA300 LAC genomic DNA with the primer pairs P2-F/P2-R and P3-F/P3-R, respectively. The products were then cloned into the modified PALC-GFP vector using ClonExpress II (C112) to generate PALC-P2::GFP and PALC-P3::GFP, respectively. The resulting vectors were transformed into USA300 LAC and Δ*narG* mutant strains, respectively, for subsequent analysis. The construction of GFP reporter vectors for N315 (PALC-P2^N315^::GFP and PALC-P3^N315^::GFP) and ATCC 29213 (PALC-P2^29213^::GFP and PALC-P3^29213^::GFP) is similar to that in USA300 LAC.

### Gene expression analysis.

Overnight cultures of the strains were diluted in TSB or basic medium RPMI 1640 (C11875500BT [Gibco]; optical density at 600 nm [OD_600_] ≈ 0.05), cultured until the indicated phases (early exponential phase [OD_600_ ≈ 0.5], midlogarithmic phase [OD_600_ ≈ 2], and early stationary phase [OD_600_ ≈ 6] for TSB; early exponential phase [OD_600_ ≈ 0.1], midlogarithmic phase [OD_600_ ≈ 0.5], and early stationary phase [OD_600_ ≈ 1.5] for RPMI 1640), and collected via centrifugation (12,000 × *g*, 2 min). For the preparation of G. mellonella hemolymph induced bacterial samples, early exponential phases of S. aureus were incubated with G. mellonella hemolymph (collected with a syringe) at 37°C for 30 min. Total RNA was extracted from cells using RNAiso plus (AKF0727A; TaKaRa), as described previously (47). For gene expression analysis, ~1 μg of total RNA was reverse-transcribed via RT-PCR using PrimeScript RT reagent kit (RR047A; TaKaRa) and CFX96 real-time system (Bio-Rad) was used for RT-qPCR. The PCR mixtures contained 5 μL of 2× RealStar Green Fast Mixture (20AE03; GenStar), 500 nM concentrations (each) of forward and reverse primers, and 1 μL of 1:3-diluted cDNA template. *pta* (gene ID 3913780) was used as the internal reference. The PCR cycling conditions for all genes (except for *hla*, *hlb*, *hlgC*, and *hld*) were as follows: 95°C for 2 min, followed by 40 cycles of 95°C for 15 s and 60°C for 30 s. Melting-curve analysis was performed for quality assurance. For *hla*, *hlb*, *hlgC*, and *hld* expression analysis, the PCR cycling conditions were as follows: 94°C for 3 min, followed by 40 cycles of 94°C for 30 s, 52°C for 30 s, and 72°C for 30 s. The relative expression of the target gene was normalized to that of the reference gene (normalized fold expression) and processed using CFX Manager 3.1 software (Bio-Rad). The RT-qPCR primers are listed in Table S2 in the supplemental material.

### Growth curves.

The target strains were cultured in TSB with shaking at 220 rpm and 37°C for ~12 h. The S. aureus cultures were diluted with fresh TSB to achieve an initial OD_600_ of 0.05 in 96-well plates containing 150 μL of TSB (200 rpm, 37°C). The OD_600_ was measured hourly using Elx800 microplate reader (Bio-Tek). Growth rate was calculated by an open-source R package, Growthcurver.

### Hemolytic activity assay.

Rabbit erythrocytes were used for the quantitative evaluation of hemolytic activity. The target strains were cultured in TSB with shaking at 220 rpm and 37°C, and the supernatants were collected in the stationary phase after centrifugation. In total, 100 μL of the supernatants were added to 900 μL of PBS containing rabbit erythrocytes at appropriate final concentrations (3% for USA300 LAC and ATCC 29213 and 15% for N315, *Δagr* mutant, and *agr* *narG* double mutant). The mixtures were incubated at 37°C for the indicated time (30 min for USA300 LAC and ATCC 29213; 12 h for N315, *Δagr* mutant, and *agr* *narG* double mutant). ddH_2_O containing 3 or 15% rabbit erythrocytes was used as the positive control, and phosphate-buffered saline (PBS) containing 3 or 15% rabbit erythrocytes was used as the negative control. The absorbance of supernatants was measured at 543 nm after centrifugation (6,000 × *g*, 1 min), and the percentage of hemolytic activity was measured relative to the positive control.

### RNA-seq.

For RNA-seq, overnight cultures of S. aureus wild type and Δ*narG* mutant strains were diluted in TSB (OD_600_ ≈ 0.05) and allowed to grow until the early exponential phase. Three samples of each strain were used for subsequent analyses. Cells were collected via centrifugation (12,000 × *g*, 2 min) and sent to Major Biotechnology Co., Ltd. (Shanghai, China), for RNA-seq. Total RNA was extracted from the S. aureus using TRIzol Reagent (15596026; Invitrogen) according to the manufacturer’s instructions and genomic DNA was removed using DNase I (D2215, TaKaRa). An RNA-seq transcriptome library was prepared following TruSeq RNA sample preparation kit from Illumina (RS-122-2001; Illumina) using 2 μg of total RNA. The rRNA depletion instead of poly(A) purification was performed by using a Ribo-Zero magnetic kit (MRZB12424; Epicenter). The data generated from the Illumina platform were used for bioinformatics analysis. All of the analyses were performed using the Majorbio free online cloud platform from Shanghai Majorbio Bio-Pharm Technology Co., Ltd. High-quality reads in each sample were mapped to the reference genome by Bowtie2 (48). We quantified gene and isoform abundances from single-end or paired-end RNA-seq data using RSEM (49). Identify differentially expressed genes using edgeR (50), DESeq2 (51), or the DESeq (52) packages. Goatools (53) was used to identify statistically significantly enriched GO terms using a Fisher exact test. After multiple testing correction, GO terms with adjusted *P* values of ≤0.05 were significantly enriched in DEGs. The raw data of RNA-seq has been uploaded to the NCBI database (BioProject PRJNA911565; GEO GSE220798).

### Fluorescence-dependent promoter activity assay.

GFP was used as a reporter gene for promoter activity analysis. Overnight cultures of the target strains were diluted in TSB (OD_600_ ≈ 0.05) and allowed to grow until reaching the indicated phase. The samples were harvested and washed twice with PBS. GFP fluorescence intensity (excitation, 488 ± 9 nm; emission, 518 ± 20 nm) was determined using CLARIOstar plate reader (BGM Labtech). The promoter activity was calculated according to the following formula: promoter activity = GFP fluorescence intensity/OD_600_.

### PSM extraction.

For extraction of PSMs, the protocol was as described previously (54, 55) with slight modification. The strains were cultured in TSB with shaking at 220 rpm and 37°C, and the supernatants were collected by centrifugation (8,000 × *g*, 30 min, 4°C) and clarified by filtration. 1-Butanol was added into the supernatants of 1.5 × 10^8^ CFU with a final concentration of 25% (vol/vol), and the samples were incubated with drastic shaking at 37°C for 2 h. Finally, the upper (butanol) phase was collected by centrifugation (12,000 × *g*, 5 min, 4°C), dried by vacuum centrifugation, and stored at −20°C until analysis. The extracts were dissolved in ddH_2_O and measured by Coomassie blue-stained 12% SDS-PAGE.

### AIP-I quantitation.

For AIP-I detection, the target strains were cultured in TSB, and the suspensions were collected until they reached the stationary phase. Samples were analyzed using an ultrahigh-pressure liquid chromatography-tandem mass spectrometer (Agilent, catalog no. 1290II-6460) with a C_18_ column (Agilent XDB-C_18_, 4.6 × 150 mm, 5 μm) at 40°C using two solvents (A: 0.1% formic acid, B: methyl alcohol). The sample injection volume was 5 μL, and the flow rate was maintained at 0.8 mL/min with 40% solvent A and 60% solvent B during analysis. For AIP-I quantitation, the concentration was calculated based on a calibration curve of the area of the ion peak versus the concentration.

### Detection of cytokines/chemokines.

For cytokine/chemokine measurements, RAW 264.7 cells (2 × 10^5^ cells/well in 12-well plates) were treated with USA300 LAC or Δ*narG* mutant strain at a multiplicity of infection (MOI) of 50 for 6 h in Dulbecco modified Eagle basic medium (C11995500BT; Gibco), and the culture supernatants were collected and stored at −80°C until analysis. Enzyme-linked immunosorbent assay (ELISA) was performed to measure the levels of cytokines/chemokines according to the manufacturer′s recommendations (12009159; Bio-Rad).

### *G. mellonella* survival assay.

Fourth-instar larvae of G. mellonella were used as target insects for bioassays. Bacterial suspensions of each isolate were cultured in TSB and allowed to grow until reaching the early exponential phase. The cells were washed three times with PBS and injected into G. mellonella with 1.5 × 10^6^ CFU in 20 μL of PBS. The larvae inoculated with PBS were used as the negative control. Each treatment was performed in triplicate with approximately 20 insects per treatment. After injection, G. mellonella larvae were incubated at 37°C for 7 days, and mortality was assessed every 24 h. Kaplan-Meier curves were used to analyze survival data, and the Mantel-Cox test was used to analyze the difference between *ΔnarG* mutant and wild type strains.

### Mouse subcutaneous abscess model.

Female BALB/c mice (outbred and immunocompetent, 6 to 8 weeks old; GemPharmatech Co., Ltd.) were used to establish the mouse subcutaneous abscess model. The mice were housed in isolated cages at an animal facility under specific-pathogen-free conditions. S. aureus isolates were cultured in TSB and allowed to grow until the early exponential phase. Then, the cells were collected and washed twice with PBS. The hair on the back of each mouse was removed using an animal shaver. The mice were anesthetized with 1% pentobarbital sodium and inoculated via subcutaneous injection into both flanks of the back with 3 × 10^7^ live S. aureus cells in 50 μL of PBS. Abscess areas were assessed as the product of the maximal length and the width of the skin lesion and were monitored daily for 7 days. The skin lesions were excised 7 days after infection and homogenized in PBS, and the number of CFU recovered from each lesion was counted via serial dilution; these cells were inoculated onto LA plates. For histological examination, the samples were fixed in a general-purpose tissue fixation solution (4% paraformaldehyde, G1101; Servicebio). Finally, the cells were sent to Wuhan Servicebio Technology for paraffin embedding and H&E (hematoxylin and eosin) staining.

### Statistical analyses.

The Shapiro-Wilk test was used to test the normality, and statistical analyses were performed using a two-tailed Student *t* test (for parametric data), a two-tailed Mann-Whitney *U* test (for nonparametric data), or a Mantel-Cox test. *P* values of <0.05 were considered to indicate statistical significance. Significance is indicated in the figures by asterisks (*, *P < *0.05; **, *P < *0.01; ***, *P < *0.001; ****, *P < *0.0001). Graphs were constructed, and analysis was performed using GraphPad Prism 8. All experiments were performed in biological triplicates. Detailed statistical data for each experiment are provided in the figure legends.

### Ethics statement.

The use and care of mice in the present study strictly followed the guidelines adopted by the Ministry of Health of the People’s Republic of China in June 2004. The protocol was approved by the Institutional Animal Care and Use Committee of the University of Science and Technology of China (USTCACUC182301015).

### Data availability.

All relevant data are either in the manuscript or in the supplemental material.
