# Supplementary material for: Nitrate Reductase NarGHJI Modulates Virulence via Regulation of agr Expression in Methicillin-Resistant Staphylococcus aureus Strain USA300 LAC
Source: Microbiol Spectr. 2023 May 18;11(3):e03596-22. doi: 10.1128/spectrum.03596-22 (PMC10269880; doi:10.1128/spectrum.03596-22)
Supplement: Supplemental file 1 — Supplemental material. Download spectrum.03596-22-s0001.pdf, PDF file, 1.6 MB [file spectrum.03596-22-s0001.pdf]

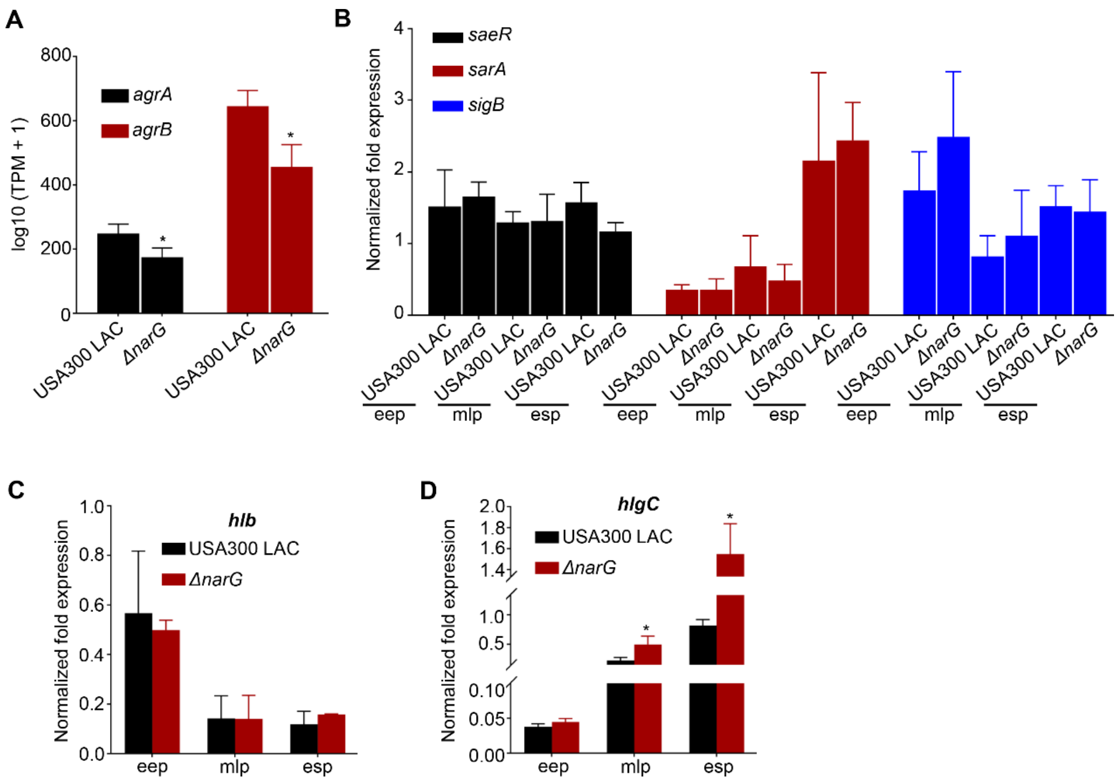

Fig S1. Expression of virulence genes. (**A**) TPM (transcripts per million) vaules of *agrA* and *agrB* from RNA-seq. *$P < 0.05$, two-tailed Mann-Whitney *U* test. (**B-D**) Determination of the expression of virulence genes *via* RT-qPCR. All experiments were performed in triplicate. Data are represented as the mean ± SD. *$P < 0.05$, two-tailed Student′s *t*-test for (**B** and **D**) and two-tailed Mann-Whitney *U* test for (**C**). eep: early exponential phase, mlp: mid-logarithmic phase, esp: early stationary phase.

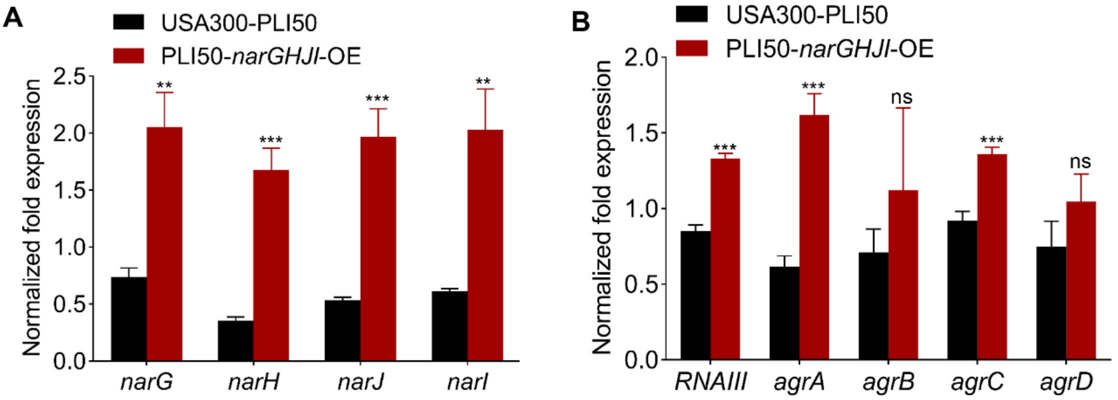

Fig S2. Overexpression of *narGHJI* promotes the expression of virulence genes. (**A**) Overexpression of *narGHJI* in USA300 LAC. (**B**) Overexpression of *narGHJI* significantly increased the expression of *RNAIII*, *agrA*, and *agrC*. For (**A** and **B**), all the samples were collected from early exponential phase of the strains. Data are represented as the mean ± SD. $^{**}P < 0.01$ and $^{***}P < 0.001$, two-tailed Student′s *t*-test. ns, not significant.

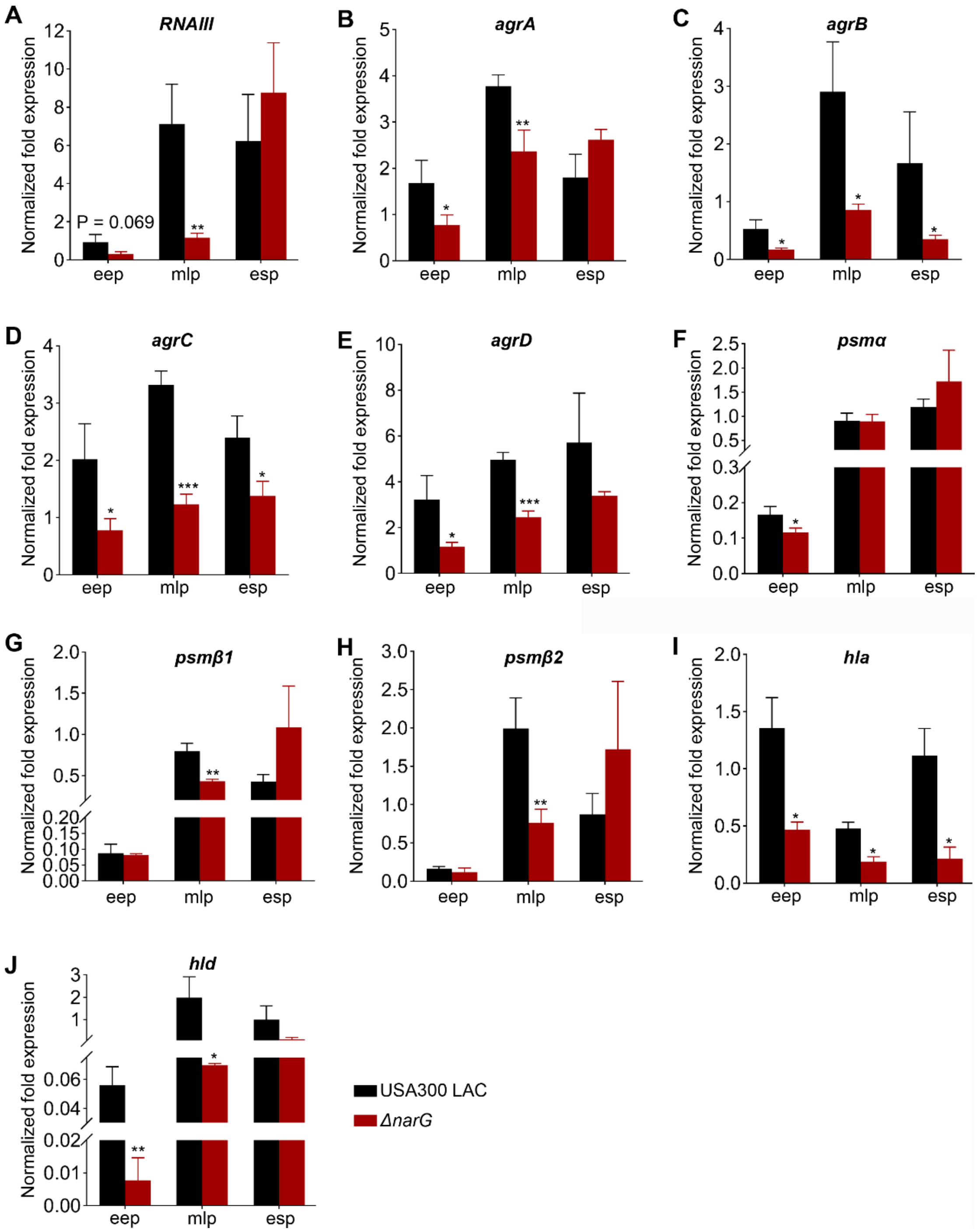

Fig S3. Determination of the expression of virulence genes by RT-qPCR. (**A-J**) *S. aureus* samples were collected from the basic medium RPMI1640. All experiments were performed in triplicate. Data are represented as the mean ± SD. *$P < 0.05$, **$P < 0.01$, and ***$P < 0.001$, two-tailed Student′s *t*-test for (**A, B, D-H,** and **J**) and two-tailed Mann-Whitney *U* test for (**C** and **I**). eep: early exponential phase, mlp: mid-logarithmic phase, esp: early stationary phase.

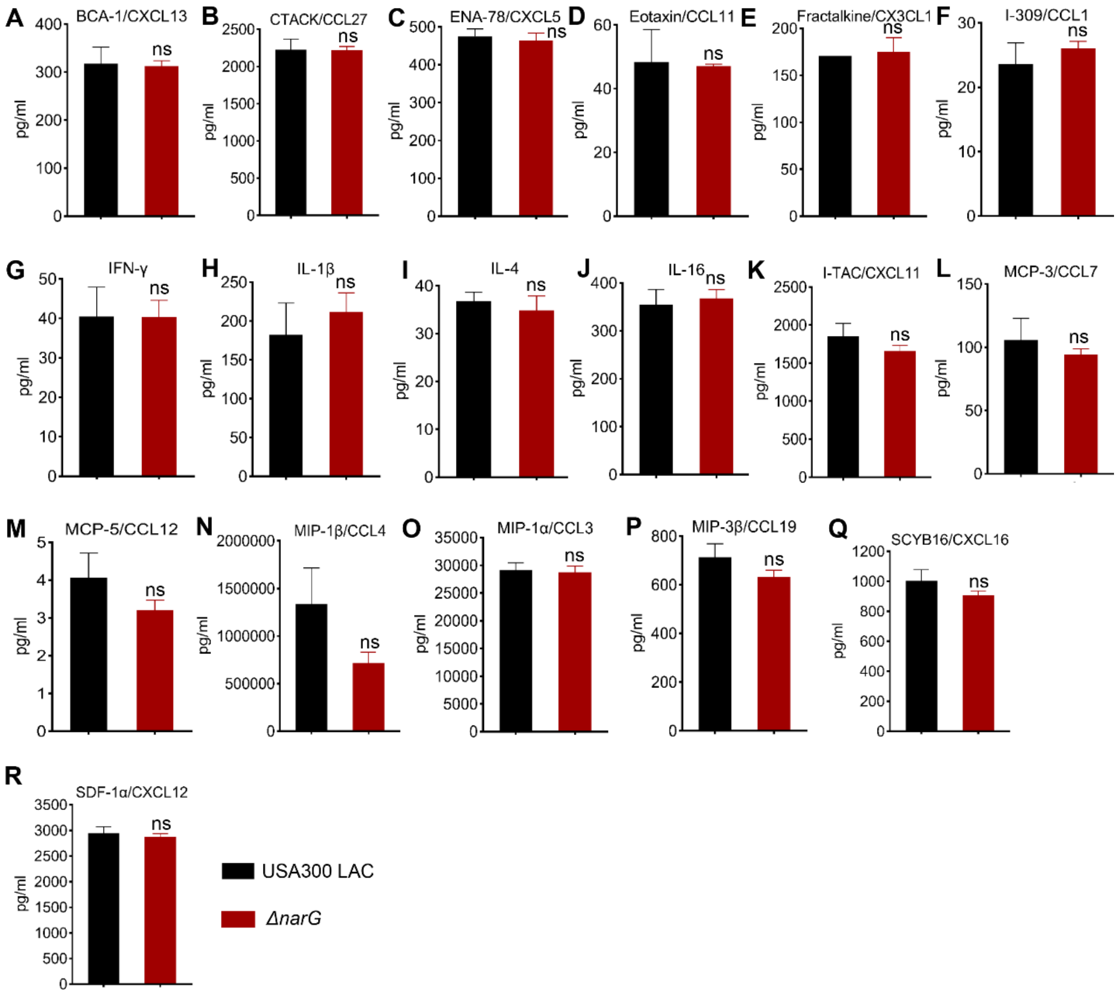

Fig S4. Determination of cytokine/chemokine levels. (**A-R**) Cytokine/chemokine production was assessed using ELISA after stimulating RAW264.7 cells with the target strains for 6 h at a MOI of 50 (n = 3). Data are represented as the mean ± SD. two-tailed Student′s *t*-test for (**B, C, F, K, M, N,** and **P**) and two-tailed Mann-Whitney *U* test for (**A, D, E, G-J, L, O, Q,** and **R**). ns, not significant.

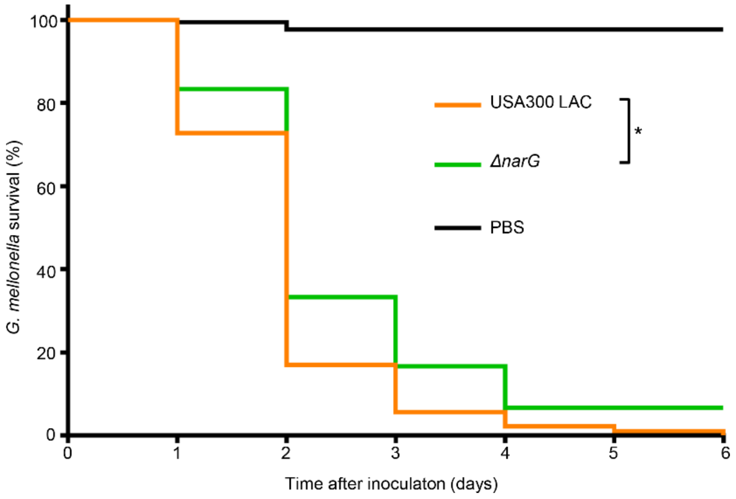

Fig S5. *narGHJI* disruption reduces the virulence of *S. aureus* in *G. mellonella* infection model. *G. mellonella* larvae were inoculated with 20 μl of PBS containing 1.5×10^6 CFUs of each target strain, and PBS was used as a negative control. Survival curves were plotted by using GraphPad Prism 8, and the Mantel-Cox test was used to analyze the difference between *ΔnarG* and USA300 LAC strains. *$P < 0.05$.

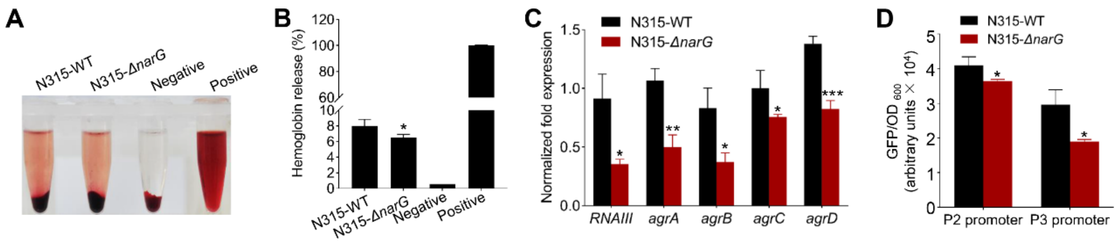

Fig S6. NarGHJI contributes to hemolytic activitie and expression of *agr* system in MRSA strain N315. (A and **B)** Hemolytic activities of the target strains. The hemolytic activities were determined by incubating the target samples with 15% rabbit erythrocytes, PBS (negative control), or ddH_2O (positive control, 100% hemolytic activity) for 12 h (**A**), and the percentage of released hemoglobin (relative to the positive control) was determined by measuring the absorbance of supernatants at 543 nm (**B**). (**C**) Determination of the expression of *agr* in the target strains. All the samples were collected from early exponential phase of the strains. Data are represented as the

mean ± SD. *$P < 0.05$, **$P < 0.01$, and ***$P < 0.001$, two-tailed Student′s *t*-test. ns, not significant. (**D**) Detection of the activities of the P2 and P3 promoters. Data are represented as the mean ± SD. *$P < 0.05$, two-tailed Student′s *t*-test. ns, not significant.

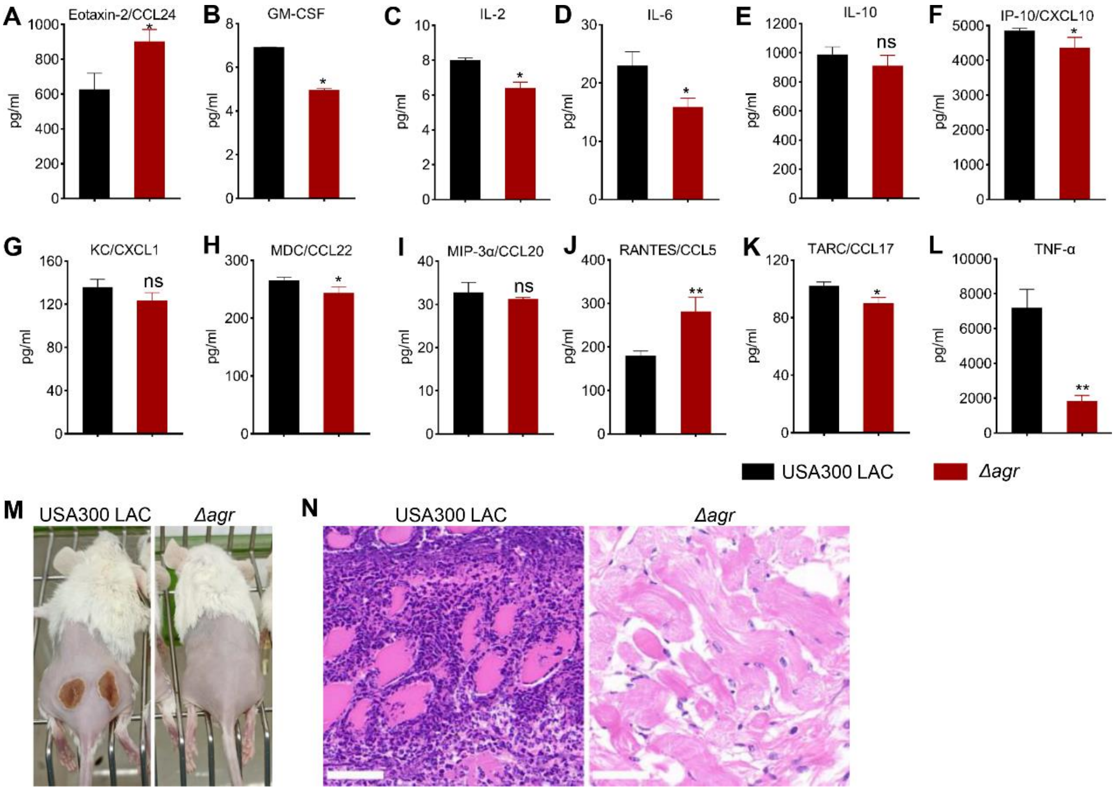

Fig S7. Cytokine/chemokine production and histological examination of *agr* mutant-infected RAW264.7 cells or mice. (**A-L**) Cytokine/chemokine production was measured using ELISA after stimulating RAW264.7 cells with the target strains for 6 h at a MOI of 50 (n = 3). Data are represented as the mean ± SD. *$P < 0.05$ and **$P < 0.01$, two-tailed Student′s *t*-test for (**A, D, E, G, H, J,** and **L**) and two-tailed Mann-Whitney *U* test for (**B, C, F, I,** and **K**). ns, not significant. (**M**) Representative abscesses at day 7 after infection. The mice were inoculated with 50 μl of PBS containing 3×10^7 CFUs of each target strain. (**N**) H&E staining of representative mouse abscesses. Scale bar = 50 μm.

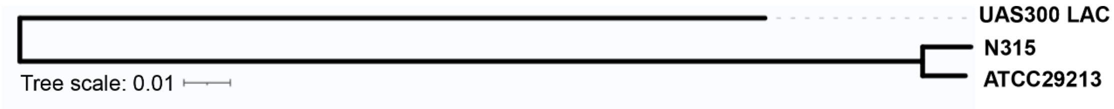

Fig S8. Phylogenetic analysis of the target strains. Phylogenetic analysis was performed by iTOL v5 (https://itol.embl.de/) based on the genomic sequences.

Table S1. Strains and plasmids used in this study.

Strain or plasmid	Description	Reference or source
***S. aureus* strains**		
RN4220	8325-4 [a]r-, initial recipient for modification of plasmids which are introduced into *S. aureus* from *E. coli*	[b]NARSA
USA300 LAC	CA-MRSA, wild-type	NARSA
narG mutant	Deletion of *narG* in USA300 LAC	This study
Com	Chromosomal complementation of *narG* mutant	This study
Δagr	Disruption of *agr* in USA300 LAC	This study
narG mutant in *Δagr*	Deletion of *narG* in *Δagr*	This study
ATCC29213	MSSA, wild-type	[c]ATCC
narG mutant in ATCC29213	Deletion of *narG* in ATCC29213	This study
N315	HA-MRSA	NARSA
narG mutant in N315	Deletion of *narG* in N315	This study
***E. coli* strains**		
TOP10	Clone host strain, F^- mcrA Δ(*mrr*-*hsd*RMS-*mcr*BC) φ80 *lacZ*Δ*M15* Δ*lac*X74 *rec*A1 *ara*Δ139 Δ(*ara-leu*)7697 *gal*U *gal*K *rps*L (Str^r) *end*A1 *nup*G	Weidi
**Plasmids**		
pBTs	Shuttle vector, temp sensitive, amp^r cm^r	This study
pBTs-LB-*narG*-RB	pBTs derivative, for *narGHJI* deletion, amp^r cm^r	This study
pBTs-*narG*-Com	pBTs derivative, for *narG* chromosomal complementation, amp^r cm^r	This study
PLI50-*narGHJI*-OE.	Overexpression of *narGHJI*, ampr cmr	This study
PALC-P2::GFP	Promoter activity assay, amp^r cm^r	This study
PALC-P3::GFP	Promoter activity assay, amp^r cm^r	This study
PALC-P2^{N315}::GFP	Promoter activity assay, amp^r cm^r	This study
PALC-P3^{N315}::GFP	Promoter activity assay, amp^r cm^r	This study
PALC-P2^{29213}::GFP	Promoter activity assay, amp^r cm^r	This study
PALC-P3^{29213}::GFP	Promoter activity assay, amp^r cm^r	This study

[a]r-, restriction system negative; kan^r, kanamycin resistant; amp^r, ampicillin resistant; cm^r, chloramphenicol resistant; str^r, streptomycin resistant.

[b]NARSA, Network on Antimicrobial Resistance in *Staphylococcus aureus*.

[c]ATCC, American Type Culture Collection.

Table S2. Primers used in this study.

Primer name	Sequence (5’ to 3’)	Comment
narG-LB-F	GCGGAATTCGAGCTCGGTACCTACCCGATAGCGATTGTCTT	*narG* disruption in USA300 LAC
narG-LB-R	CATTTTCATCTTCATCCAGCATGATCTAGGATCGACCAATTCCCA	*narG* disruption in USA300 LAC, N315, and ATCC29213
narG-RB-F	TTTAATGGGAATTGGTCGATCCTAGATCATGCTGGATGAAGATGA	*narG* disruption in USA300 LAC, N315, and ATCC29213
narG-RB-R	CTTGCATGCCTGCAGGTCGACATAGAACCAACTTGTTCCGT	*narG* disruption in in USA300 LAC
narG-LB-R (com)	CATCGCTGGAATAGGTGTAAATCCTGCGATTCGATCTGGACCAT	*narG* chromosomal complementation
narG-RB-F (com)	GGATTTACACCTATTCCAGCGATGTCAATGATTAGTTATGCAGC	*narG* chromosomal complementation
narG-MCS-F	TTGCTACTATGCAGACAGGT	*narG* mutant/complemented strain screening
narG-MCS-R	GCACGTTGCAAAATCGCTTC	*narG* mutant/complemented strain screening
agr-LB-F	GCGGAATTCGAGCTCGGTACCTTACAAGAGGTTGAACAAGC	*agr* disruption
agr-LB-R	TTTTAGTGAATTTGTTCACTGTGTCCAAAGTTGCAGCGATGGATT	*agr* disruption
agr-RB-F	AATAAAATCCATCGCTGCAACTTTGGACACAGTGAACAAATTCAC	*agr* disruption
agr-RB-R	CTTGCATGCCTGCAGGTCGACGTCATTGGAACTAATAGCAC	*agr* disruption
narG-LB-F (N315)	GCGGAATTCGAGCTCGGTACCTATCCGATAGCGATTGTCTT	*narG* disruption in N315
narG-RB-R (N315)	CTTGCATGCCTGCAGGTCGACATAGAACCAACTTGTTCCAT	*narG* disruption in N315
narG-LB-F (29213)	GCGGAATTCGAGCTCGGTACCTATCCGATAGCGATTGTCTT	*narG* disruption in ATCC29213
narG-RB-R (29213)	CTTGCATGCCTGCAGGTCGACATAGAACCAACTTGTTCCAT	*narG* disruption in ATCC29213
narGHJI-OE-F	AGCTCGGTACCCGGGGATCCTCGTGCAGATGCTAATACAG	Overexpression of *narGHJI*
narGHJI-OE-R	AGGTCGACTCTAGAGGATCCTTATTACACTTTATTCTTACGGT	Overexpression of *narGHJI*
pta-F	AAAGCGCCAGGTGCTAAATTAC	Internal reference in RT-qPCR analysis
pta-R	CTGGACCAACTGCATCATATCC	Internal reference in RT-qPCR analysis
*narG*ex-F	TATGCAGCAGGTGCTCGATT	RT-qPCR analysis of *narG*
*narG*ex-R	ACCTTTATAGCGGACTTCAG	RT-qPCR analysis of *narG*
*narH*ex-F	AACACTACAAAGGTGGTTGG	RT-qPCR analysis of *narH*

*narH*ex-R	CTGAATACGCTCTAGCAACT	RT-qPCR analysis of *narH*
*narJ*ex-F	GGATATATGGCACAACAGTT	RT-qPCR analysis of *narJ*
*narJ*ex-R	GCTAACATTTGCCCACGTTC	RT-qPCR analysis of *narJ*
*narI*ex-F	TTTGTCGCAGGTCATATTGC	RT-qPCR analysis of *narI*
*narI*ex-R	CACACCCACTGCATCCATCC	RT-qPCR analysis of *narI*
RNAIII-F	GCCATCCCAACTTAATAACC	RT-qPCR analysis of *RNAIII* in USA300 LAC, N315, and ATCC29213
RNAIII-R	ACGATAGCTTACATGCTAGA	RT-qPCR analysis of *RNAIII* in USA300 LAC, N315, and ATCC29213
agrA-F	CGTGGCAGTAATTCAGTGTA	RT-qPCR analysis of *agrA* in USA300 LAC
agrA-R	TATGGCGATTGACGACAAAG	RT-qPCR analysis of *agrA* in USA300 LAC
agrB-F	TGCACATGGTGCACATGCAC	RT-qPCR analysis of *agrB* in USA300 LAC
agrB-R	AATAAGTCGCACAGGAATGG	RT-qPCR analysis of *agrB* in USA300 LAC
agrC-F	CTCGGATGAAGCTAAAGTAA	RT-qPCR analysis of *agrC* in USA300 LAC
agrC-R	AATCATGACGGAACTTGCGC	RT-qPCR analysis of *agrC* in USA300 LAC
agrD-F	CTTATTTTTTGATTTTATTACTGGG	RT-qPCR analysis of *agrD* in USA300 LAC
agrD-R	CGTGTAATTGTGTTAATTCTTTTGG	RT-qPCR analysis of *agrD* in USA300 LAC
psmα-F	GTATCATCGCTGGCATCA	RT-qPCR analysis of *psmα* in USA300 LAC
psmα-R	AAGACCTCCTTTGTTTGTTATG	RT-qPCR analysis of *psmα* in USA300 LAC
psmβ1-F	CGCAATTAAAGATACCGTAACTGCAGC	RT-qPCR analysis of *psmβ1* in USA300 LAC
psmβ1-R	ACCTAATAAACCTACGCC	RT-qPCR analysis of *psmβ1* in USA300 LAC
psmβ2-F	TGGACTAGCAGAAGCAATCG	RT-qPCR analysis of *psmβ2* in USA300 LAC
psmβ2-R	CTAGTAAACCCACACCGTTA	RT-qPCR analysis of *psmβ2* in USA300 LAC
hla-F	CCCGGTATATGGCAATCAAC	RT-qPCR analysis of *hla* in USA300 LAC
hla-R	GGTAGTCATCACGAACTCGT	RT-qPCR analysis of *hla* in USA300 LAC
hlb-F	GATAGCAACAAAGGCTTTGT	RT-qPCR analysis of *hlb* in USA300 LAC
hlb-R	GGTCGCCACCTATATATACC	RT-qPCR analysis of *hlb* in USA300 LAC
hlgC-F	CTCTTGCCAATCCGTTATTA	RT-qPCR analysis of *hlgC* in USA300 LAC

hlgC-R	GTTCTAGAGCTAATGAATCC	RT-qPCR analysis of *hlgC* in USA300 LAC
hld-F	GAGTTGTTTAATTTTAAG	RT-qPCR analysis of *hld* in USA300 LAC
hld-R	TTTTAGTGAATTTGT	RT-qPCR analysis of *hld* in USA300 LAC
saeR-F	CGCCTTAACTTTAGGTGCAG	RT-qPCR analysis of *saeR* in USA300 LAC
saeR-R	ATAGGGACTTCGTGACCATT	RT-qPCR analysis of *saeR* in USA300 LAC
sarA-F	ATGGTCACTTATGCTGACAA	RT-qPCR analysis of *sarA* in USA300 LAC
sarA-R	GGTTGTTTGTAGTTTAAATG	RT-qPCR analysis of *sarA* in USA300 LAC
sigB-F	GGTGCCATAAATAGATTCGA	RT-qPCR analysis of *sigB* in USA300 LAC
sigB-R	CACCGATTACAGTAGGTACT	RT-qPCR analysis of *sigB* in USA300 LAC
agrA$^{15/13}$-F	CCAGCAGAATTAAGAACTCG	RT-qPCR analysis of *agrA* in N315 and ATCC29213
agrA$^{15/13}$-R	CGGTTATCTAAATGGGCAAT	RT-qPCR analysis of *agrA* in N315 and ATCC29213
agrB$^{15/13}$-F	CGCTATAATGCACATGGTGC	RT-qPCR analysis of *agrB* in in N315 and ATCC29213
agrB$^{15/13}$-R	CGTTGCTGCAGGTGCATAAA	RT-qPCR analysis of *agrB* in in N315 and ATCC29213
agrC$^{15/13}$-F	ACAAGTTCAAACGGTGATAG	RT-qPCR analysis of *agrC* in in N315 and ATCC29213
agrC$^{15/13}$-R	TGACATAATCATGACGGAAC	RT-qPCR analysis of *agrC* in in N315 and ATCC29213
agrD$^{15/13}$-F	TAGCTAAAGCAATCGGAATTG	RT-qPCR analysis of *agrD* in in N315 and ATCC29213
agrD$^{15/13}$-R	AAATTCGTTAATTCAGCGGG	RT-qPCR analysis of *agrD* in in N315 and ATCC29213
P2-F	AAACGACGGCCAGTGAATTCTTAAACAACTCATCAACTATT	Activity detection of P2 promoter in USA300 LAC, N315, and ATCC29213
P2-R	ACCATGGTGGCGACGAATTCAAACTGGTCAATTTTATTATC	Activity detection of P2 promoter in USA300 LAC
P3-F	AAACGACGGCCAGTGAATTCTGTCATTATACGATTTAGTA	Activity detection of P3 promoter in USA300 LAC
P3-R	ACCATGGTGGCGACGAATTCTTAAACAACTCATCAACTATT	Activity detection of P3 promoter in USA300 LAC, N315, and ATCC29213
P2$^{15/13}$-R	ACCATGGTGGCGACGAATTCAAACTGGTCAATTTTGTTATC	Activity detection of P2 promoter in N315 and ATCC29213
P3$^{15/13}$-F	AAACGACGGCCAGTGAATTCTGTTATTATACGATTTAGTAC	Activity detection of P3 promoter in N315 and ATCC29213

Table S3. Differentially expressed genes from RNA-seq.

Gene_ID	*Gene name*	Gene description	FC (*ΔnarG*/USA300 LAC)	P-value	Significant	Regulate
JYB01_RS04550	*JYB01_RS04550*	ATP phosphoribosyltransferase	0.267	5.39275E-12	yes	down
JYB01_RS04095	*JYB01_RS04095*	protein VraX	0.285	8.76002E-29	yes	down
JYB01_RS04545	*JYB01_RS04545*	ATP phosphoribosyltransferase regulatory subunit	0.294	7.47545E-13	yes	down
JYB01_RS09720	*lrgB*	antiholin-like protein LrgB	0.307	5.96335E-18	yes	down
JYB01_RS04560	*JYB01_RS04560*	histidinol-phosphate aminotransferase family protein	0.319	1.37506E-20	yes	down
JYB01_RS04555	*JYB01_RS04555*	histidinol dehydrogenase	0.321	7.3149E-20	yes	down
sRNA0022			0.333	3.90368E-44	yes	down
JYB01_RS02390	*JYB01_RS02390*	argininosuccinate synthase	0.365	4.85714E-25	yes	down
JYB01_RS04565	*hisB*	imidazoleglycerol-phosphate dehydratase HisB	0.372	1.27244E-13	yes	down
JYB01_RS02480	*JYB01_RS02480*	Na^+/H^+ antiporter family protein	0.4	8.95359E-26	yes	down
JYB01_RS02395	*argH*	argininosuccinate lyase	0.424	1.94012E-16	yes	down
JYB01_RS09715	*lrgA*	antiholin-like murein hydrolase modulator LrgA	0.424	6.74791E-09	yes	down
JYB01_RS04575	*JYB01_RS04575*	1-(5-phosphoribosyl)-5-((5-phosphoribosylamino)methylideneamino)imidazole-4-carboxamide isomerase	0.432	8.30218E-16	yes	down
JYB01_RS04580	*hisF*	imidazole glycerol phosphate synthase subunit HisF	0.432	9.85757E-12	yes	down
JYB01_RS04780	*argF*	ornithine carbamoyltransferase	0.432	1.71499E-06	yes	down
JYB01_RS05680	*cntK*	histidine racemase CntK	0.436	3.19131E-10	yes	down
JYB01_RS04585	*JYB01_RS04585*	bifunctional phosphoribosyl-AMP cyclohydrolase/phosphoribosyl-ATP diphosphatase HisIE	0.472	2.2184E-09	yes	down
sRNA0028			0.476	6.55984E-13	yes	down
JYB01_RS05235	*farE*	fatty acid efflux MMPL transporter FarE	0.485	6.36674E-13	yes	down
JYB01_RS05210	*cwrA*	cell wall inhibition responsive protein CwrA	0.488	3.11797E-20	yes	down
JYB01_RS12365	*JYB01_RS12365*	ABC transporter permease subunit	0.489	1.13529E-11	yes	down

JYB01_RS00130	*JYB01_RS00130*	aspartate kinase	0.489	5.76702E-11	yes	down
JYB01_RS05690	*cntM*	staphylopine dehydrogenase CntM	0.492	6.0357E-08	yes	down
JYB01_RS12370	*JYB01_RS12370*	amino acid ABC transporter ATP-binding protein	0.502	5.73878E-13	yes	down
JYB01_RS04570	*hisH*	imidazole glycerol phosphate synthase subunit HisH	0.502	2.34747E-06	yes	down
JYB01_RS00110	*dapD*	2%2C3%2C4%2C5-tetrahydropyridine-2%2C6-dicarboxylate N-acetyltransferase	0.509	3.06137E-10	yes	down
JYB01_RS07715	*hrcA*	heat-inducible transcription repressor HrcA	0.514	2.82616E-14	yes	down
JYB01_RS00105	*JYB01_RS00105*	amidohydrolase	0.516	3.54556E-10	yes	down
JYB01_RS13285	*JYB01_RS13285*	hypothetical protein	0.518	0.000292852	yes	down
JYB01_RS04785	*arcD*	arginine-ornithine antiporter	0.52	1.7862E-05	yes	down
JYB01_RS13045	*JYB01_RS13045*	AzlD domain-containing protein	0.521	1.27337E-12	yes	down
JYB01_RS02015	*JYB01_RS02015*	hypothetical protein	0.522	1.92065E-08	yes	down
JYB01_RS05685	*cntL*	D-histidine (S)-2-aminobutanoyltransferase CntL	0.522	1.44296E-07	yes	down
sRNA0021			0.529	2.73061E-09	yes	down
JYB01_RS14125	*JYB01_RS14125*	YjiH family protein	0.542	1.06375E-20	yes	down
JYB01_RS13920	*JYB01_RS13920*	MFS transporter	0.542	2.55063E-09	yes	down
JYB01_RS00125	*JYB01_RS00125*	aspartate-semialdehyde dehydrogenase	0.545	5.90154E-10	yes	down
JYB01_RS12825	*JYB01_RS12825*	sodium-dependent transporter	0.561	2.15598E-13	yes	down
JYB01_RS05200	*copZ*	copper chaperone CopZ	0.561	1.59231E-11	yes	down
JYB01_RS06075	*JYB01_RS06075*	nitrate reductase subunit alpha	0.563	1.70612E-07	yes	down
JYB01_RS02845	*JYB01_RS02845*	hypothetical protein	0.563	4.33264E-06	yes	down
JYB01_RS00715	*JYB01_RS00715*	hypothetical protein	0.564	2.78778E-12	yes	down
JYB01_RS03170	*JYB01_RS03170*	ABC transporter ATP-binding protein	0.564	0.001621418	yes	down
JYB01_RS06240	*JYB01_RS06240*	response regulator transcription factor	0.567	4.75491E-06	yes	down
JYB01_RS00115	*JYB01_RS00115*	4-hydroxy-tetrahydrodipicolinate reductase	0.572	4.22498E-10	yes	down
JYB01_RS06255	*JYB01_RS06255*	ABC transporter permease	0.573	7.18739E-06	yes	down

JYB01_RS05345	*JYB01_RS05345*	hypothetical protein	0.574	0.000413184	yes	down
JYB01_RS02815	*JYB01_RS02815*	amino acid transporter	0.577	6.04773E-06	yes	down
JYB01_RS06235	*JYB01_RS06235*	DUF3021 domain-containing protein	0.587	6.03397E-06	yes	down
JYB01_RS04775	*arcA*	arginine deiminase	0.588	0.001656444	yes	down
JYB01_RS10500	*JYB01_RS10500*	hypothetical protein	0.594	4.5063E-05	yes	down
JYB01_RS09000	*sbnC*	staphyloferrin B biosynthesis protein SbnC	0.595	0.004358335	yes	down
JYB01_RS14140	*JYB01_RS14140*	ABC transporter substrate-binding protein	0.601	2.25557E-07	yes	down
JYB01_RS10655	*JYB01_RS10655*	delta-lysin family phenol-soluble modulin	0.601	2.5826E-07	yes	down
JYB01_RS04515	*JYB01_RS04515*	S-adenosyl-l-methionine hydroxide adenosyltransferase family protein	0.601	0.000342806	yes	down
JYB01_RS04520	*JYB01_RS04520*	ECF-type riboflavin transporter substrate-binding protein	0.604	0.000424889	yes	down
JYB01_RS09920	*JYB01_RS09920*	cell wall-active antibiotics response protein	0.606	1.38013E-08	yes	down
JYB01_RS00100	*JYB01_RS00100*	alanine racemase	0.607	2.80105E-05	yes	down
JYB01_RS09500	*uhpT*	hexose-6-phosphate:phosphate antiporter	0.607	3.22033E-05	yes	down
JYB01_RS14130	*JYB01_RS14130*	iron chelate uptake ABC transporter family permease subunit	0.609	1.47798E-07	yes	down
JYB01_RS01295	*JYB01_RS01295*	beta-class phenol-soluble modulin	0.609	0.001342814	yes	down
JYB01_RS07725	*dnaK*	molecular chaperone DnaK	0.612	5.72317E-08	yes	down
JYB01_RS06260	*JYB01_RS06260*	ABC transporter ATP-binding protein	0.615	7.12085E-06	yes	down
sRNA0001			0.615	0.001773291	yes	down
JYB01_RS10625	*groL*	chaperonin GroEL	0.616	6.41426E-08	yes	down
JYB01_RS00650	*JYB01_RS00650*	glycerol-3-phosphate dehydrogenase/oxidase	0.618	3.38079E-11	yes	down
JYB01_RS05295	*JYB01_RS05295*	sterile alpha motif-like domain-containing protein	0.619	6.59908E-06	yes	down
JYB01_RS09570	*JYB01_RS09570*	acyl CoA:acetate/3-ketoacid CoA transferase	0.619	0.002978355	yes	down
JYB01_RS10675	*JYB01_RS10675(agrA)*	response regulator transcription factor	0.62	1.31008E-06	yes	down
JYB01_RS02475	*JYB01_RS02475*	PaaI family thioesterase	0.622	1.67417E-10	yes	down

JYB01_RS00120	*JYB01_RS00120*	4-hydroxy-tetrahydrodipicolinate synthase	0.623	1.76852E-06	yes	down
JYB01_RS10660	*agrB*	accessory gene regulator AgrB	0.627	1.74059E-07	yes	down
JYB01_RS07250	*JYB01_RS07250*	amino acid permease	0.627	7.04088E-07	yes	down
JYB01_RS06130	*JYB01_RS06130*	MarR family transcriptional regulator	0.629	1.64065E-09	yes	down
JYB01_RS04265	*JYB01_RS04265*	ribosomal L7Ae/L30e/S12e/Gadd45 family protein	0.63	0.000883347	yes	down
JYB01_RS06125	*JYB01_RS06125*	Hsp20/alpha crystallin family protein	0.633	3.98515E-08	yes	down
JYB01_RS10670	*JYB01_RS10670*	GHKL domain-containing protein	0.637	3.32261E-06	yes	down
JYB01_RS08870	*JYB01_RS08870*	hypothetical protein	0.638	3.92754E-10	yes	down
JYB01_RS00420	*rpmG*	50S ribosomal protein L33	0.64	3.74281E-11	yes	down
JYB01_RS01000	*JYB01_RS01000*	acyl carrier protein	0.642	8.00753E-06	yes	down
JYB01_RS09455	*JYB01_RS09455*	M23 family metallopeptidase	0.646	6.20265E-08	yes	down
JYB01_RS02565	*JYB01_RS02565*	DUF3055 domain-containing protein	0.652	2.11318E-06	yes	down
JYB01_RS10630	*groES*	co-chaperone GroES	0.655	1.73617E-05	yes	down
JYB01_RS09860	*sgtB*	monofunctional peptidoglycan glycosyltransferase SgtB	0.657	1.9353E-06	yes	down
JYB01_RS10200	*JYB01_RS10200*	hypothetical protein	0.659	0.00444983	yes	down
JYB01_RS00415	*rpsN*	30S ribosomal protein S14	0.66	0.000981634	yes	down
JYB01_RS00660	*JYB01_RS00660*	aquaporin family protein	0.661	4.65379E-08	yes	down
JYB01_RS00460	*JYB01_RS00460*	homoserine dehydrogenase	0.662	1.04301E-05	yes	down
JYB01_RS05715	*JYB01_RS05715*	ABC transporter ATP-binding protein	0.666	0.000386142	yes	down
JYB01_RS10925	*JYB01_RS10925*	thiol-disulfide oxidoreductase DCC family protein	1.5	9.6823E-06	yes	up
JYB01_RS10330	*JYB01_RS10330*	HK97 gp10 family phage protein	1.506	0.00158111	yes	up
JYB01_RS14310	*JYB01_RS14310*	TIGR01741 family protein	1.509	0.002532251	yes	up
JYB01_RS09595	*JYB01_RS09595*	nitric oxide dioxygenase	1.51	6.48122E-06	yes	up
JYB01_RS00030	*sucB*	dihydrolipoyllysine-residue succinyltransferase	1.515	2.78455E-06	yes	up
JYB01_RS10520	*JYB01_RS10520*	DUF2829 domain-containing protein	1.52	0.001861033	yes	up
JYB01_RS05255	*clpL*	ATP-dependent Clp protease ATP-binding subunit ClpL	1.523	7.79754E-06	yes	up

JYB01_RS05180	*JYB01_RS05180*	phytoene/squalene synthase family protein	1.523	0.000321853	yes	up
JYB01_RS09755	*rbsD*	D-ribose pyranase	1.524	0.000263799	yes	up
JYB01_RS02715	*JYB01_RS02715*	winged helix-turn-helix domain-containing protein	1.535	0.002781265	yes	up
JYB01_RS07315	*JYB01_RS07315*	uroporphyrinogen-III synthase	1.539	8.81735E-07	yes	up
JYB01_RS03910	*adhP*	alcohol dehydrogenase AdhP	1.542	3.24039E-06	yes	up
JYB01_RS02805	*JYB01_RS02805*	organic hydroperoxide resistance protein	1.55	1.12277E-05	yes	up
JYB01_RS05185	*JYB01_RS05185*	NAD(P)/FAD-dependent oxidoreductase	1.554	6.02962E-05	yes	up
JYB01_RS07000	*JYB01_RS07000*	hypothetical protein	1.564	0.00609108	yes	up
JYB01_RS06895	*JYB01_RS06895*	type Z 30S ribosomal protein S14	1.569	7.4644E-06	yes	up
JYB01_RS08180	*JYB01_RS08180*	DUF1672 domain-containing protein	1.576	0.006691985	yes	up
JYB01_RS12855	*aaa*	autolysin/adhesin Aaa	1.59	4.79927E-11	yes	up
JYB01_RS07165	*JYB01_RS07165*	DUF2273 domain-containing protein	1.615	2.26107E-08	yes	up
JYB01_RS01305	*JYB01_RS01305*	hypothetical protein	1.618	0.00033834	yes	up
JYB01_RS06350	*JYB01_RS06350*	DUF805 domain-containing protein	1.624	6.00037E-09	yes	up
JYB01_RS04755	*isaB*	immunodominant staphylococcal antigen IsaB	1.633	2.03124E-09	yes	up
JYB01_RS10325	*JYB01_RS10325*	hypothetical protein	1.642	3.84529E-05	yes	up
JYB01_RS11650	*JYB01_RS11650*	twin-arginine translocase TatA/TatE family subunit	1.649	3.74842E-09	yes	up
JYB01_RS12620	*JYB01_RS12620*	hypothetical protein	1.65	1.89021E-07	yes	up
JYB01_RS10395	*JYB01_RS10395*	hypothetical protein	1.656	0.003077936	yes	up
JYB01_RS13895	*JYB01_RS13895*	ABC transporter permease	1.666	0.000765824	yes	up
JYB01_RS05170	*JYB01_RS05170*	NAD(P)/FAD-dependent oxidoreductase	1.687	1.24825E-06	yes	up
JYB01_RS05175	*crtQ*	4%2C4'-diaponeurosporenoate glycosyltransferase	1.692	8.81773E-09	yes	up
JYB01_RS10510	*JYB01_RS10510*	phage antirepressor KilAC domain-containing protein	1.745	1.72437E-06	yes	up
JYB01_RS01730	*JYB01_RS01730*	DNA-dependent RNA polymerase auxiliary subunit epsilon family protein	1.746	5.95457E-09	yes	up
JYB01_RS10260	*JYB01_RS10260*	phage holin	1.748	0.008162488	yes	up

JYB01_RS04315	*rpmG*	50S ribosomal protein L33	1.764	1.59273E-06	yes	up
JYB01_RS10375	*JYB01_RS10375*	HNH endonuclease	1.765	0.000973069	yes	up
JYB01_RS09960	*ftnA*	H-type ferritin FtnA	1.784	5.55803E-16	yes	up
JYB01_RS14205	*JYB01_RS14205*	excalibur calcium-binding domain-containing protein	1.795	5.01429E-07	yes	up
JYB01_RS10495	*JYB01_RS10495*	DUF771 domain-containing protein	1.811	0.001246417	yes	up
JYB01_RS10525	*JYB01_RS10525*	helix-turn-helix transcriptional regulator	1.813	0.001595155	yes	up
JYB01_RS04750	*JYB01_RS04750*	hypothetical protein	1.817	1.95798E-06	yes	up
JYB01_RS08800	*norB*	multidrug efflux MFS transporter NorB	1.838	4.48277E-05	yes	up
JYB01_RS10385	*JYB01_RS10385*	DUF1514 family protein	1.935	0.005420028	yes	up
JYB01_RS10465	*JYB01_RS10465*	recombinase RecT	1.944	2.12832E-06	yes	up
JYB01_RS13270	*JYB01_RS13270*	HNH endonuclease	1.975	6.07181E-06	yes	up
JYB01_RS10470	*JYB01_RS10470*	AAA family ATPase	2.043	4.55291E-06	yes	up
JYB01_RS09520	*pflB*	formate C-acetyltransferase	2.062	2.15963E-10	yes	up
JYB01_RS13140	*JYB01_RS13140*	helix-turn-helix domain-containing protein	2.103	1.78161E-06	yes	up
JYB01_RS05445	*JYB01_RS05445*	ABC transporter permease	2.12	8.15676E-09	yes	up
JYB01_RS05450	*JYB01_RS05450*	ATP-binding cassette domain-containing protein	2.185	2.36699E-09	yes	up
JYB01_RS10450	*JYB01_RS10450*	DnaD domain-containing protein	2.228	3.73823E-08	yes	up
JYB01_RS10455	*ssb*	single-stranded DNA-binding protein	2.272	2.03626E-06	yes	up
JYB01_RS10420	*JYB01_RS10420*	DUF1024 family protein	2.293	0.000689934	yes	up
JYB01_RS09525	*pflA*	pyruvate formate lyase-activating protein	2.35	1.08567E-12	yes	up
JYB01_RS06120	*JYB01_RS06120*	NarK/NasA family nitrate transporter	2.521	1.24244E-16	yes	up
JYB01_RS08785	*ald*	alanine dehydrogenase	2.531	1.73328E-12	yes	up
JYB01_RS08795	*JYB01_RS08795*	amino acid permease	2.532	6.46344E-09	yes	up
JYB01_RS06060	*JYB01_RS06060*	NAD(P)/FAD-dependent oxidoreductase	2.571	8.36988E-33	yes	up
JYB01_RS06070	*cobA*	uroporphyrinogen-III C-methyltransferase	2.576	1.45952E-24	yes	up
JYB01_RS06065	*nirD*	nitrite reductase small subunit NirD	2.672	1.27145E-23	yes	up

JYB01_RS10480	*JYB01_RS10480*	DUF1108 family protein	2.692	2.42102E-05	yes	up
sRNA0009			2.72	1.38276E-09	yes	up
JYB01_RS08790	*tdcB*	bifunctional threonine ammonia-lyase/L-serine ammonia-				
		lyase TdcB	2.968	1.88089E-12	yes	up
sRNA0012			3.052	0.007223845	yes	up
JYB01_RS06055	*JYB01_RS06055*	sirohydrochlorin chelatase	3.301	7.5305E-38	yes	up

Table S4. Multilocus sequence typing of the target strains.

Strain	ST	*arcC*	*aroE*	*glpF*	*gmK*	*pta*	*tpi*	*yqiL*
USA300 LAC	8	3	3	1	1	4	4	3
ATCC29213	5	1	4	1	4	12	1	10
N315	5	1	4	1	4	12	1	10
